# Efficacy and immunogenicity of a single dose of human papillomavirus vaccine compared to multidose vaccination regimens or no vaccination: An updated systematic review of evidence from clinical trials

**DOI:** 10.1016/j.jvacx.2024.100486

**Published:** 2024-04-16

**Authors:** Hilary S. Whitworth, Sandra Mounier-Jack, Edward M. Choi, Katherine E. Gallagher, Natasha Howard, Helen Kelly, Gladys Mbwanji, Aimée R Kreimer, Partha Basu, Ruanne Barnabas, Mélanie Drolet, Marc Brisson, Deborah Watson-Jones

**Affiliations:** a Faculty of Infectious and Tropical Diseases, London School of Hygiene and Tropical Medicine, London, United Kingdom; b Faculty of Public Health and Policy, London School of Hygiene and Tropical Medicine, London, United Kingdom; c Saw Swee Hock School of Public Health, National University of Singapore and National University Health System, Singapore; d Mwanza Intervention Trials Unit, National Institute of Medical Research, Mwanza, Tanzania; e National Cancer Institute, National Institute of Health, Bethesda, MD, United States; f International Agency for Research on Cancer, World Health Organization, Lyon, France; g Division of Infectious Diseases, Massachusetts General Hospital and Harvard Medical School, Boston, United States; h Department of Social and Preventive Medicine, Laval University, Québec, Canada

**Keywords:** Human papillomavirus, Vaccine, Dosage, Efficacy, Immunogenicity

## Abstract

**Objectives:**

This study systematically reviewed the published literature from clinical trials on the efficacy and immunogenicity of single-dose HPV vaccination compared to multidose schedules or no HPV vaccination.

**Methods:**

Four databases were searched for relevant articles published from Jan-1999 to Feb-2023. Articles were assessed for eligibility for inclusion using pre-defined criteria. Relevant data were extracted from eligible articles and a descriptive quality assessment was performed for each study. A narrative data synthesis was conducted, examining HPV infection, other clinical outcomes and immunogenicity responses by dose schedule.

**Results:**

Fifteen articles reporting data from six studies (all in healthy young females) were included. One article was included from each of three studies that prospectively randomised participants to receive a single HPV vaccine dose versus one or more comparator schedule(s). The other 12 articles reported data from three studies that randomised participants to receive multidose HPV vaccine (or control vaccine) schedules; in those studies, some participants failed to complete their allocated schedule, and evaluations were conducted to compare participants who actually received one, two or three doses. Across all efficacy studies, the incidence or prevalence of HPV16/18 infection was very low among HPV-vaccinated participants, regardless of the number of doses received; with no evidence for a difference between dose groups. In immunogenicity studies, HPV16/18 antibody seropositivity rates were high among all HPV-vaccinated participants. Antibody levels were significantly lower with one dose compared to two or three doses, but levels with one dose were stable and sustained to 11 years post-vaccination.

**Conclusions:**

Results from this review support recent World Health Organization recommendations allowing either one- or two-dose HPV vaccination in healthy young females. Longer-term efficacy and immunogenicity data from ongoing studies are awaited. Randomised trials of single-dose HPV-vaccination are urgently needed in other populations, e.g. boys, older females and people with HIV.

## Highlights

• This study reviewed the literature from clinical trials on the efficacy and immunogenicity of single-dose HPV vaccination.
• Rates of HPV16/18 infection were low among all HPV vaccine recipients and did not qualitatively differ by dose group.
• Almost all HPV vaccine recipients generated a detectable HPV16/18 IgG antibody response.
• HPV16/18 antibody levels with one dose were significantly lower than with two or three doses but were stable to 11 years post-vaccination.
• This review supports recent WHO recommendations allowing one- or two-dose HPV vaccination in healthy young females.

## Introduction

Cervical cancer is the second most common cancer among women <65 years globally and was responsible for over 340,000 deaths in 2020 [1]. The burden of cervical cancer is greatest in low and middle-income countries (LMICs), where ∼90 % of cases occur. The highest cervical cancer incidence rates worldwide are observed in Sub-Saharan Africa [1]. Almost all cervical cancer cases are caused by persistent infection of the cervix with oncogenic genotypes of Human Papillomavirus (HPV).

HPV vaccination of adolescent girls is one of three key pillars of the Global Strategy for cervical cancer elimination, which was adopted by the World Health Assembly in 2020 [2]. The strategy calls for 90 % of girls worldwide to be vaccinated against HPV by 15 years of age by 2030 [2]. However, this figure was just 15 % in 2019 [3], and coverage has fallen since the start of the COVID-19 pandemic [4]. The low global HPV vaccine coverage to date stems largely from delayed/non-introduction of HPV vaccine into national vaccination programmes in many countries, as well as suboptimal coverage in many countries that have introduced the vaccine; in 2019, ∼70 % of girls worldwide lived in countries that had not yet introduced HPV vaccination [3]. Furthermore, there are major disparities in HPV vaccine introduction according to countries’ economic levels. By March 2022, <50 % of LMICs had introduced HPV vaccination, compared with almost 90 % of high-income countries (HICs) [5].

A major barrier to HPV vaccine introduction has been the substantial costs and logistical challenges of implementing the original licensed multidose schedules [6]. Additionally, temporary HPV vaccine supply constraints in recent years delayed vaccine introduction in some countries [7]. However, in 2022, the World Health Organization (WHO) amended its recommendations to allow either one-dose or two-dose HPV vaccination in people aged 9–20 years [8]. A two-dose schedule is recommended for people aged ≥21 years, and at least two doses (ideally three) are recommended for those aged ≥9 years with HIV. This change in recommendation follows a decade of evidence from non-randomized single-dose recipients in RCTs, observational studies and, more recently, prospectively-randomised clinical trials, suggesting that one dose of HPV vaccine elicits a robust and sustained antibody response that is sufficient to provide similar protection against cervical HPV infection as a two-dose or three-dose vaccine schedule [9], [10], [11], [12], [13].

In 2018, we conducted a systematic literature review of evidence from clinical trials on the efficacy and immunogenicity of single-dose HPV vaccination compared to no vaccination or to multidose schedules [11]. Given recent changes in WHO dosing recommendations and increasing numbers of countries considering the introduction of (or switch to) single-dose HPV vaccination, we aimed to update our review, combining earlier evidence on the immunogenicity and efficacy of single-dose HPV vaccination together with robust new evidence that has emerged in the past five years.

## Methods

### Research questions and design

This systematic literature review aimed to address two research questions:

1. “Does one dose of HPV vaccine elicit similar efficacy against HPV infection and associated clinical outcomes, and similar immune responses, as a two-dose or three-dose schedule?”
2. “Does one dose of HPV vaccine provide protection against HPV infection and associated clinical outcomes when compared to no HPV vaccination, i.e., is one dose better than not vaccinating?”

The review was designed to capture data on single-dose HPV vaccination versus multidose schedules or no HPV vaccination from clinical trials. This includes data from clinical trials that specifically randomised participants to receive one dose of HPV vaccine versus a comparator. However, it also includes data from clinical trials of multidose HPV vaccine schedules where some participants received only one vaccine dose due to non-completion of their originally assigned vaccine regimen. In the latter scenario, data were considered to be observational.

Our original systematic review was conducted in 2018 [11]. Since that time, we periodically updated the review to identify and synthesise relevant data as they became available.

The methods used to conduct our review have been described in detail previously [11]. Below, we briefly summarise these methods and describe updates conducted since our earlier publication. The review was registered in PROSPERO (registration ID 110162) and is reported according to Preferred Reporting Items for Systematic Reviews and Meta-Analyses (PRISMA).

### Search strategy

Our previously published systematic review included articles published from 01-January-1999 to 14-August-2018 [11]. We have since updated the included evidence to 04-February-2023 through three subsequent searches.

Database searches were conducted as described previously [11]. In brief, one author (HSW) searched for relevant publications in four scientific databases using Medical subject heading (MeSH) and non-MeSH terms for ‘human papillomavirus’, ‘vaccines’, ‘immunogenicity or efficacy/effectiveness’, and ‘dosage’. Supplementary Table 1 details the databases searched and search syntax used. Search results were exported to Endnote and duplicates were excluded. HSW additionally conducted backward reference searching of all included articles, and of relevant review articles identified in the same database searches (Supplementary Table 2).

### Eligibility screening and data extraction

Search results were screened according to pre-defined eligibility criteria (Supplementary Table 3). First, titles and abstracts were double-screened based on selected eligibility criteria by two of six reviewers (EMC, GM, HSW, KEG, NH, and SMJ), with ineligible articles excluded. Second, full texts of remaining articles were double-screened against the full eligibility criteria, with final eligibility confirmed by consensus across duplicate reviews. Discordant reviews were resolved either through discussion or by a third reviewer as the tie-breaker.

Data were extracted by HSW using a standardised form, as described previously [11]. Tabulated results were independently verified by a second author (HK or EMC).

### Quality assessment

KEG or HSW conducted a descriptive quality assessment and synthesis for each of the included studies, evaluating risk of selection bias, confounding, retention or survival bias, and misclassification of the exposure or outcome. The assessment also evaluated the appropriateness of statistical analyses and the generalisability of study results. The potential impact of any confounding, bias or misclassification was considered, particularly examining for instances that may artificially increase vaccine efficacy observed with one vaccine dose or decrease efficacy observed with multiple doses.

### Data analysis

HSW conducted an initial narrative data synthesis, as described previously [11].

Clinical outcome measures evaluated in our review were driven by those reported in the included studies. In primary analyses, we examined HPV16/18 infections. In secondary analyses, we examined the spectrum of vaccine-type infections among recipients of the 4vHPV and 9vHPV vaccines (Gardasil® and Gardasil-9®, respectively, manufactured by Merck & Co), as well as HPV31/33/45 infections (to evaluate the potential for cross-protection) among recipients of the 2vHPV vaccine (Cervarix®, manufactured by GSK Biologicals) and the 4vHPV vaccine. Data tables present infection event and numerator data extracted directly from each article. However, reporting of measures of occurrence were standardised in our review through computation of prevalence and incidence risk, with exact 95 % confidence intervals (CI), using the extracted data. Extracted data were additionally used to calculate unadjusted prevalence ratios (PRs) and risk ratios (RRs) for one HPV vaccine dose versus two doses, three doses or no HPV vaccination. P-values were obtained for each comparison using the two-sided Fisher’s exact test.

For this paper, immunogenicity assessments focused on binding antibody seropositivity, concentrations and avidity for the relevant HPV genotypes. In primary analyses, we examined HPV16 and HPV18 responses. In secondary analyses, we examined other vaccine-type responses among recipients of the 4vHPV and 9vHPV vaccines. As above, tables present extracted data on numbers of participants testing seropositive for HPV antibodies and numerator data, whilst seropositivity proportions with exact 95 %CI were recalculated specifically for this review to standardise reporting. Antibody concentrations and antibody avidity (as geometric mean [GM] concentrations or avidity index with 95 %CI) are presented as shown in the original articles.

All analyses were performed using Stata, version 16.0 (Stata, College Station, Texas). Data pooling and *meta*-analysis were not conducted due to heterogeneity in study designs, methods and outcome measures.

## Results

### Search results

**Fig. 1:**
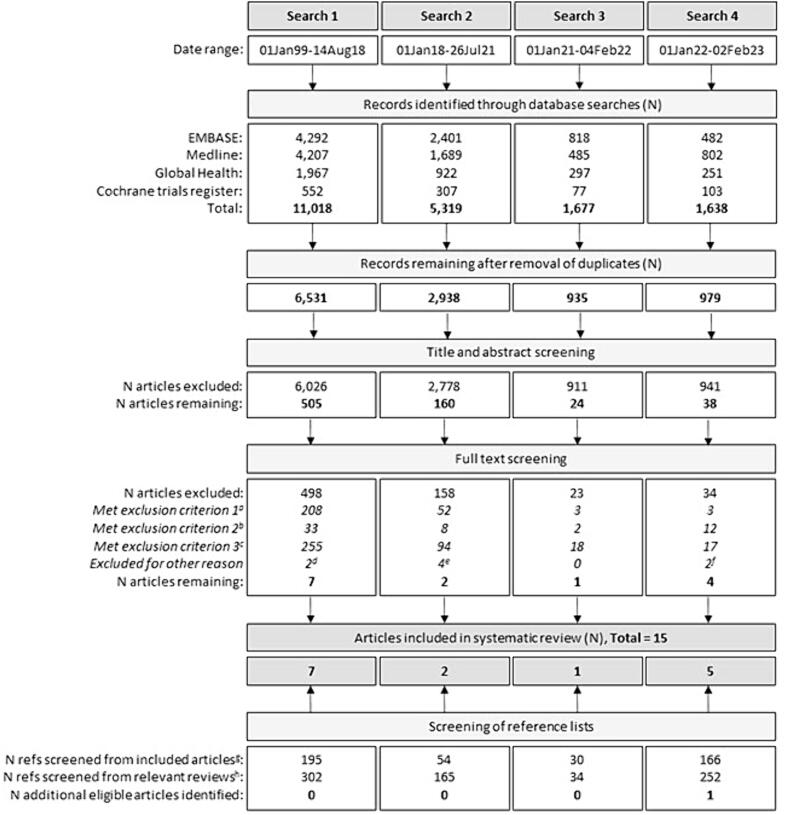
Systematic review flow chart, including the original search and the three updated searches. ^a^Exclusion criterion 1: Article does not describe a research study with human participants who received a prophylactic HPV vaccine through a clinical trial setting. ^b^Exclusion criterion 2: Article does not present post-vaccination efficacy or humoral immunogenicity data. ^c^Exclusion criterion 3: Article does not present data for one HPV vaccine dose versus no HPV vaccination, or versus a multidose HPV vaccination schedule, at the same timepoint(s). ^d^One article was a correction to a previously published study (that was already identified for inclusion in the review) [47]; the other summarised data from previously published studies but did not include any new data or analyses [48]. ^e^Two articles had already been identified through the original August 2018 search [16], [19], one described an evaluation of serological assays for measuring antibody responses to one-dose HPV vaccination [49], and one described a trial of the Innovax 2vHPV vaccine but provided insufficient information on one-dose vaccination to calculate infection measures such as proportions and risk ratios [33]. ^f^Two articles were not accessible online or through our institution’s library [50], [51]. The authors of the articles were contacted and requested to provide the full-text papers but did not respond. ^g^Reference lists were screened from the 14 eligible articles that were identified from the database searches. ^h^Reference lists were screened from 22 relevant review articles, which are listed in Supplementary Table 2.

Four systematic literature searches were conducted between August-2018 and February-2023, identifying 15 articles eligible for inclusion in our review (Fig. 1; Table 1). Seven articles were identified from the first search [14], [15], [16], [17], [18], [19], [20], two from the second [21], [22], one from the third [23] and five from the fourth [12], [13], [24], [25], [26].

**Table 1:** Summary of articles presenting data on one HPV vaccine dose versus no HPV vaccination or a multidose schedule. Articles are grouped by their originating study (indicated by shading) and presented in order of date of publication.

Abbreviations: 2vHPV, bivalent HPV [vaccine]; 4vHPV, quadrivalent HPV [vaccine]; 9vHPV, nonavalent HPV [vaccine]; CVT, Costa Rica vaccine trial; d, dose; DNA, deoxyribonucleic acid; FU, follow-up; GSK, GlaxoSmithKline; HAV, hepatitis A vaccine; HIC, high-income countries; HPV, human papillomavirus; IARC, International Agency for Research on Cancer; Immuno: immunogenicity; LMIC, low- and middle-income countries; M/m, month/months; N, number; RCT, randomized controlled trial; US, United States; y, years.

^a^HPV16/18 DNA + refers to a positive HPV16 and/or HPV18 result on PCR/genotyping using cervical or vaginal samples. HPV16/18 sero + refers a positive HPV16 and/or HPV18 antibody result using serum or plasma. Baseline refers to pre-vaccination.

^b^The CVT enrolled 3,730 HPV-vaccinated and 3,736 HAV-vaccinated participants. At Y4, 2,919 of the HPV vaccinated participants were enrolled into the CVT LTFU, along with a new control group of 2,836 unvaccinated participants. Numbers of participants included in the analyses in each of these CVT/CVT LTFU articles were as follows: Kreimer 2011 [14]: 3,575 HPV-vaccinated and 3,578 HAV-vaccinated participants; Safaeian 2018 [16]: 2,449 HPV vaccinated and 2,382 unvaccinated participants; Kreimer 2020 [21]: 1,539 HPV-vaccinated and 1,783 unvaccinated participants; Tsang 2020 [22]: 2,974 HPV-vaccinated, 3,315 HAV-vaccinated and 2,619 unvaccinated participants.

^c^In these articles, HPV16/18 DNA and serostatus are reported among the participants included in analyses (i.e., not among all participants enrolled).

^d^Tsang 2022 [24], Kreimer 2015 [17], Sankaranarayanan 2016 [18], Joshi 2023 [25] and Watson-Jones 2022 [13] only present baseline serostatus and/or DNA status for HPV16 and HPV18 separately. In Kreimer 2015, results are presented on the proportion of participants who were seropositive and/or DNA positive at baseline. In Joshi 2023, results for baseline serostatus are presented among all HPV vaccinated combined, not by arm.

^e^Of the 13,049 HPV-vaccinated and 13,061 HAV-vaccinated participants enrolled in the CVT and the PATRICIA trial, 12,159 and 12,194, respectively, were included in the post-hoc analysis.

^f^The IARC India Study enrolled 17.729 participants. Girls were eligible for annual collection of cervical samples (and thus inclusion in efficacy evaluations) from 6 months after delivery of a baby or 12 months after marriage; whichever was earlier. Two years into the study, a control group of 1,574 unvaccinated women was recruited. Numbers of participants included in efficacy evaluations in each of these articles were as follows: Sankaranarayanan 2016: 2,649 HPV-vaccinated participants; Sankaranarayanan 2018: 5,655 HPV-vaccinated and 1,481 unvaccinated participants; Basu 2021: 9,183 HPV-vaccinated and 1,484 unvaccinated participants. Immunological evaluations were conducted in an immunogenicity sub-cohort, which was selected by convenience sampling (with differing numbers included per time point).

^g^Notably, seropositivity cut-offs were calculated based on the MFI values of serum samples obtained from participants at baseline. Cut-off values were defined after allowing for 5% seropositivity among the total baseline samples.

^h^As well as the annual HPV testing described above, married HPV-vaccinated women were invited for cervical cancer screening when they reached 25 years of age, The first unvaccinated control group was used for comparison in annual HPV testing and were also eligible for cervical cancer screening when they turned 25 years of age. The second unvaccinated control group was recruited for comparison in cervical cancer screening only (not annual HPV testing).

^i^The follow-up duration was not reported. However, the participants were 18–25 years old at the time of sample collection, compared to 10–18 years old at the time of vaccination.

^j^This study only included participants who were HPV16 seropositive at baseline; results are not presented on baseline HPV18 serostatus.

^k^Overall, 661 (29 %) participants tested HPV16/18 antibody or DNA positive at enrolment or at 3 months post-vaccination. However, the breakdown by arm or time point (enrolment vs M3) is not reported in the article.

^l^The 2vHPV vaccine was administered at M0, M1 and M6; the 9vHPV vaccine was administered at M0, M2 and M6.

The 15 eligible articles present data from six different studies (all of healthy girls and young women) (Table 1). Two of these studies – the KEN-SHE trial (from Kenya) [12] and the DoRIS trial (from Tanzania) [13] – are ongoing prospectively-designed randomised controlled clinical trials (RCTs) comparing efficacy or immunogenicity outcomes following a single HPV vaccine dose versus a control vaccine or versus multidose HPV vaccine schedules. One other prospectively randomised study (from the United States [US]) was a small pilot study comparing one HPV vaccine dose to no HPV vaccination among women who were HPV16 seropositive at baseline [20].

The remaining three studies – the Costa Rica Vaccine Trial (CVT) and its long-term follow-up (LTFU), the multi-country PATRICIA trial and the IARC India Vaccine Study – originally randomised participants to receive two-dose or three-dose HPV vaccine schedules (versus a control vaccine in the CVT and the PATRICIA trial). The relevant articles identified for inclusion in our systematic review present data on efficacy or immunogenicity outcomes among participants who completed and failed to complete their allocated vaccine schedule (i.e., with a proportion of the latter group receiving only one HPV vaccine dose) [14], [15], [16], [17], [18], [19], [21], [22], [23], [24], [25], [26]. The included data from these three studies are considered to be observational as allocation to the dosing schedule arms (i.e., one dose versus multidose schedules or no vaccination) was according to what participants actually received rather than what they were allocated to receive. For the CVT and the IARC India Study, the numerous articles present data from the same girls and women across different follow-up timepoints. One article presents combined data from the CVT and the PATRICIA trial.

The six studies represented in this review, and the 15 articles from which data are derived, are described in more detail in the Supplementary Results. An in-depth narrative review of the quality assessment performed for each study is provided in Supplementary Table 4.

### HPV infection results

#### Cervical HPV16/18 infections

Ten articles included data on prevalent, incident and/or persistent cervical HPV16/18 infections following one dose of HPV vaccine versus a comparator schedule; five from the CVT or CVT LTFU [14], [16], [21], [22], [24], one from the CVT/PATRICIA combined analysis [17], three from the IARC India Study [18], [19], [23] and one from the KEN-SHE trial [12]. For each of these articles, Supplementary Table 5 summarises the sampling performed, the laboratory methods used for HPV detection, and the infection outcome measures reported and their definitions. Table 2 presents HPV16/18 infection results for one dose versus each comparator schedule for studies evaluating the 2vHPV vaccine. Table 3 presents the same results for studies evaluating the 4vHPV or 9vHPV vaccines. Data extend to 1.5 years post dose one vaccination for the 9vHPV vaccine, 10 years for the 4vHPV vaccine, and 11 years for the 2vHPV vaccine.

**Table 2:** HPV16/18 infection results from articles comparing one dose of the 2vHPV vaccine to either no HPV vaccination or a multidose schedule.

Abbreviations: 2vHPV, bivalent HPV [vaccine]; CI, confidence interval; CVT, Costa Rica Vaccine Trial; HPV, human papillomavirus; GSK, GlaxoSmithKline; IQR, interquartile range; LTFU, long-term follow-up; m, month; N, number of participants in group; NC, not calculated; PR, prevalence ratio; RR, risk ratio; SD, standard deviation; UTC, unable to compute; y, years.

^a^Definitions of infection outcomes used in each study are provided in Supplementary Table 5. All endpoints refer to cervical infections unless stated otherwise.

^b^Results are shown only for two-dose arms where participants received dose one at day 0 and dose two at day 180.

^c^Results are shown for one-dose control vaccine arms for Kreimer 2011 [14], Kreimer 2015 [17], Tsang 2022 [24] (all HAV vaccine) and Barnabas 2023 [12] (meningococcal vaccine), and unvaccinated control arms for Safaeian 2018 [16] (persistent infection only), Kreimer 2020 [21] and Tsang 2020 [22]. For Kreimer 2011, Kreimer 2015 and Tsang 2022, comparison of the one-dose HPV vaccine arm with the one-dose HAV (rather than multidose HAV) arm minimizes the potential for selection bias due to differences in follow-up.

^d^Proportions (%), unadjusted RRs and PRs, 95%CI and two-sided Fisher’s exact p-values were calculated by the authors of the systematic review using data provided in the included articles. In most cases, the 95%CI for proportions calculated by the authors of this review matched those reported in the included studies. Where they do differ, the 95%CI calculated in this review are generally wider than those reported in the articles.

^e^In studies where participants were not specifically randomised to receive one HPV vaccine dose versus either no HPV vaccination or multidose HPV vaccination, RRs and PRs calculated for one versus two or three doses must be interpreted with caution because of potential for selection bias due to differences in follow-up between the groups.

^f^STATA does not compute a p-value using Fisher’s exact test where both numerators are 0.

^g^Kreimer 2020 [21] and Tsang 2020 [22] are sister articles, both presenting efficacy results to Y11 in CVT participants. Kreimer 2020 focuses on HPV16/18; Tsang 2020 focuses on cross-protection, but also presents HPV16/18 results. Thus, there is a lot of overlap in the HPV16/18 results presented in this table from the two articles.

^h^Median follow-up time is presented separately in the article for Y0-4 of CVT and the subsequent long-term follow-up (to Y11). Median follow-up time was 4.5 years for all arms in the CVT and ranged from 6.3 years in the unvaccinated control arm to 6.7 years in the 3-dose arm in the long-term follow-up.

^i^IQR or SD were not reported for this study.

^j^Results are shown for new infections detected at Y9 that persisted for at least 150 days.

^k^Mean, median, IQR, or SD were not reported for these studies.

^l^Results are presented in the article for HPV16 infections only, not HPV18. The article presented HPV16 infection results for participants who were seronegative versus seropositive for antibodies to HPV16 at enrolment. Results shown in this table are for women who were seronegative for antibodies to HPV16 at enrolment.

**Table 3:** HPV16/18 infection results from articles comparing one dose of the 4vHPV or 9vHPV vaccine to either no HPV vaccination or a multidose schedule.

Abbreviations: 4vHPV, quadrivalent HPV [vaccine]; 9vHPV, nonavalent HPV [vaccine]; CI, confidence interval; HPV, human papillomavirus; IARC, International Agency for Research on Cancer; IQR, interquartile range; m, month; N, number of participants in group; NC, not calculated; PR, prevalence ratio; RR, risk ratio; UTC, unable to compute; y, years.

^a^Definitions of infection outcomes used in each study are provided in Supplementary Table 5. All endpoints refer to cervical infections unless stated otherwise.

^b^Results are shown only for two-dose arms where participants received dose one at day 0 and dose two at day 180.

^c^Results are shown for a one-dose control vaccine arm for Barnabas 2022 [12] (meningococcal vaccine), and unvaccinated control arms for Sankaranarayanan 2018 [19], Basu 2021 [23] and Gheit 2023 [26]. No control arm was included in Sankaranarayanan 2016 [18].

^d^Proportions (%), unadjusted RRs and PRs, 95%CI and two-sided Fisher’s exact p-values were calculated by the authors of the systematic review using data provided in the included articles. In most cases, the 95%CI for proportions calculated by the authors of this review matched those reported in the included studies. Where they do differ, the 95%CI calculated in this review are generally wider than those reported in the articles.

^e^In studies where participants were not specifically randomised to receive one HPV vaccine dose versus either no HPV vaccination or multidose HPV vaccination, RRs and PRs calculated for one versus two or three doses must be interpreted with caution because of potential for selection bias due to differences in follow-up between the groups.

^f^Mean, median, IQR, or SD were not reported for these studies.

^g^Sankaranarayanan 2016 [18] aimed to measure persistent infection but did not detect any persistent infections in any arm.

^h^STATA does not compute a p-value using Fisher’s exact test where both numerators are 0.

Across all studies that provided relevant infection data, the frequency of cervical HPV16/18 infection following HPV vaccination was very low, regardless of the number of doses received, and substantially lower than the frequency of infection in control groups. The KEN-SHE trial did not include multidose HPV vaccine arms; however, up to year (Y)1.5, only two endpoints of six-month persistent HPV16/18 infection were detected among participants who received a single HPV vaccine dose, one in the 2vHPV vaccine arm and one in the 9vHPV vaccine arm, giving an incidence risk of just 0.2 % (95 %CI 0.0–1.1 %) per arm. For comparison, the incidence of infection was significantly higher in the meningococcal-vaccinated control group (7.6 %, 95 %CI 5.4–10.4 %). In their paper, the authors reported vaccine efficacy (VE) of 97.5 % (95 %CI 81.6–99.7 %) for one dose of the 2vHPV vaccine against incident six-month persistent HPV16/18 infection, and 97.5 % (95 %CI 81.7–99.7 %) for one dose of the 9vHPV vaccine.

Similarly, by Y4, there were no six-month persistent HPV16/18 infections detected among single-dose 2vHPV vaccine recipients from the CVT and the PATRICIA trial, and the incidence in this group (0.0 %, 95 %CI 0.0–1.9 %) was significantly lower than in the single-dose control (Hepatitis A vaccine [HAV]) group (8.0 %, 95 %CI 4.5–12.8 %). Data were not reported on persistent infections occurring to the Y11 timepoint in the CVT LTFU, but a similar pattern was seen for prevalent and one-time incident HPV16/18 infections. Due to less frequent sampling, the IARC India Study examined 10- or 12-month persistent infections. However, as in the other studies, the incident risk for 10-month persistent HPV16/18 infection up to 10 years post 4vHPV vaccination was significantly lower in the one-dose arm (0.0 %, 95 %CI 0.0–0.3 %) than in the unvaccinated control group (2.5 %, 95 %CI 1.7–3.6 %).

Among HPV-vaccinated participants of the CVT, the PATRICIA trial and the IARC India Study, there was no evidence of a difference in the frequency of prevalent, incident or persistent HPV16/18 infection by the number of doses received at any timepoint. In the combined CVT/PATRICIA analysis, the incidence of six-month persistent HPV16/18 infection up to Y4 was 0.3 % (95 %CI 0.0–1.9 %) in the one-dose arm, 0.7 % (0.2–1.7 %) in the two-dose arm, and 1.0 % (0.8–1.2 %) in the three-dose arm. In their paper, the authors reported similar VE for one (96.6 %, 95 % CI 81.7–99.8 %), two (89.7 %, 95 %CI 73.3–96.9 %) and three (89.1 %, 95 %CI 86.8–91.0 %) 2vHPV vaccine doses against incident six-month persistent HPV16/18 infection. Findings were similar for one-time incident and prevalent infection occurring up to Y11 in the CVT LTFU. In the IARC India Study, the incidence of 10-month persistent HPV16/18 infection up to Y10 was 0.0 % (95 %CI 0.0–0.3 %) in the one-dose arm and 0.1 % (0.0–0.4 %) in each of the two-dose and three-dose arms. The authors of the IARC India Study calculated VE adjusted for several potential confounders (described in Supplementary Table 4); in that analysis, they found no difference in VE against persistent HPV16/18 infection by the number of vaccine doses received (one-dose: 95.4 %, 95 % CI 85.0–99.9 %; two-dose: 93.1 %, 95 %CI 77.3–99.8 %; three-dose: 93.3 %, 95 %CI 77.5–99.7 %).

#### Cervical 4vHPV and 9vHPV vaccine-type HPV infections

The article from the KEN-SHE trial [12] and two articles from the IARC India Study [18], [23] present data on other vaccine-type cervical HPV infections following 4vHPV or 9vHPV vaccination. As observed for HPV16/18, the frequency of cervical vaccine-type HPV infections was very low among HPV-vaccinated participants, and lower compared to control participants, regardless of the number of doses received (Supplementary Table 6). In the KEN-SHE trial, the incidence of six-month persistent HPV16/18/31/33/45/52/58 infection occurring to Y1.5 was 1.2 % (95 %CI 0.3–3.1 %) in the one-dose 9vHPV vaccine arm and 10.0 % (95 %CI 6.8–14.0 %) in the control arm. The VE for one-dose 9vHPV vaccination reported by the authors was 88.9 % (95 % CI 68.5–96.1 %). Similarly, in the IARC India Study, the incidence of 10-month persistent HPV6/11/16/18 infection occurring to Y10 was 0.1 % (95 %CI 0.0–0.3 %) in the one-dose 4vHPV vaccine arm and 2.8 % (95 %CI 1.9–3.8 %) in the control arm.

In the IARC India Study, there was no evidence for a difference in the frequency of vaccine-type infections according to the number of vaccine doses received. Up to Y10, the incidence of 10-month persistent HPV6/11/16/18 infection was 0.1 % in each of the one-dose (95 %CI 0.0–0.3 %), two-dose (95 %CI 0.0–0.4 %) and three-dose (95 %CI 0.0–0.5 %) arms. Adjusted for multiple potential confounders, the authors reported no difference in VE by the number of vaccine doses received (one-dose: 93.4 %, 95 % CI 81.1–99.1 %; two-dose: 93.7 %, 95 %CI 79.8–99.8 %; three-dose: 90.3 %, 95 %CI 71.9–98.5 %).

#### Cervical HPV31/33/45 infections

Two articles from the CVT or CVT LTFU [16], [22], one from the CVT/PATRICIA combined analysis [17], and three from the IARC India Study [18], [19], [23], report on HPV31/33/45 infections following 2vHPV or 4vHPV vaccination.

In the combined CVT/PATRICIA analysis, the incidence of six-month persistent HPV31/33/45 infection up to Y4 was similar across 2vHPV vaccinated arms that received one dose (3.1 %, 95 %CI 1.4–5.8 %), two doses (2.9 %, 95 %CI 1.7–4.6 %) and three doses (2.3 %, 95 %CI 2.1–2.7 %) (Supplementary Table 7). The equivalent point estimate was almost two-times higher in the control arm (5.9 %, 95 %CI 3.3–9.6 %) compared to the one-dose HPV vaccine arm, but 95 %CI were wide and overlapping. Findings were similar up to Y11 in the CVT LTFU.

In the IARC India Study, there was no evidence of a difference in the incidence of 10-month persistent HPV31/33/45 infection up to Y10 across any of the arms (HPV vaccinated and control). However, the incidence of one-time HPV31/33/45 infection was similar across the one-dose (4.8 %, 95 %CI 4.0–5.6 %), two-dose (4.1 %, 95 %CI 3.3–5.0 %) and three-dose arms (4.3 %, 95 %CI 3.4–5.2 %), and significantly higher in the control arm (10.0 %, 95 %CI 8.5–11.7).

In each of these articles, the authors did not report any evidence for differences in VE against persistent HPV31/33/45 infection across dose groups (after adjusting for potential confounders in the IARC India Study), though 95 %CI were generally very wide (data not shown).

#### Oral HPV infections

The sub-study to the IARC India Study examined oral HPV infections among a sub-group of participants who received one, two or three doses of the 4vHPV vaccine, and a sub-group of the unvaccinated control participants [26].

Point estimates for the prevalence of oral HPV16/18 infection were lowest in the two-dose arm (1.1 %, 95 %CI 0.1–3.8 %), followed by the three-dose (2.5 %, 95 %CI 1.1–4.8 %) and one-dose arms (2.5 %, 95 %CI 0.8–5.6 %), followed by the control arm (4.5 %, 95 % 1.9–8.6 %) (Table 3). Similar results were seen for HPV6/11/16/18 prevalence (Supplementary Table 6). However, estimates are derived from very low infection event counts, thus resulting in wide and overlapping 95 %CI; and there appeared to be imbalance in the prevalence of non vaccine-type HPV infections across dose arms.

### Cervical cancer screening results

One article from the IARC India Study reported on HPV infection results and cervical intraepithelial neoplasia (CIN) and invasive cervical cancer (ICC) cases from the cervical cancer screening cohort [23]. Seven of 4,819 women vaccinated with the 4vHPV vaccine (0.1 %, 95 %CI 0.1–0.3 %) (one from the three-dose arm, four from the two-dose arm and two from the one-dose arm) tested positive for HPV16/18 infection using a screening test (that was different from the assay to detect HPV for incident and persistent infections) by Y10, compared with 63 of 4,626 unvaccinated women (1.4 %, 95 %CI 1.0–1.7 %). One case of HPV16/18-associated CIN1 was detected among vaccinated women (in a two-dose participant), compared with five cases of CIN1, two cases of CIN2 and one case of CIN3 among unvaccinated women.

### Immunogenicity results

#### Antibody seropositivity and concentrations

Eight articles present data on HPV16 and HPV18 binding antibody seropositivity and/or GM concentrations (GMCs) following one dose of HPV vaccine versus a comparator schedule; three from the CVT or CVT LTFU [15], [16], [21], three from the IARC India Study [18], [19], [25], one from the US pilot study [20], and one from the DoRIS trial [13]. Two of the articles from the IARC India Study also present data on HPV6 and HPV11 responses [18], [25]. For each of the eight articles, Supplementary Table 8 summarises the sampling performed, the laboratory methods used, and the immunogenicity outcome measures reported. Table 4 presents HPV16 and HPV18 seropositivity proportions and antibody GMCs for one dose versus multidose schedules for studies evaluating the 2vHPV vaccine. Table 5 presents the same data for studies evaluating the 4vHPV or 9vHPV vaccine; and Supplementary Table 9 presents the corresponding HPV6 and HPV11 immunogenicity data. Data extend up to two years post dose one vaccination for the 9vHPV vaccine, 10 years for the 4vHPV vaccine, and 11 years for the 2vHPV vaccine.

**Table 4:** HPV16 and 18 seropositivity and geometric mean antibody level results from articles evaluating one versus two or three doses of the 2vHPV vaccine.

Reference/study	Time point	N seropositive^a^/N participants (% Seropositive, 95 %CI^b^)			GM concentrations (95 %CI)		
		3 doses	2 doses^c^	1 dose	3 doses	2 doses^c^	1 dose
**HPV16**							
Safaeian 2013/CVT [15]^d^	M0	18/120 (15.0, 9.1–22.7)	−	6/78 (7.7, 2.9–16.0)	<LOD	<LOD	<LOD
	M6	−	−	−	724 EU/ml	102 EU/ml	145 EU/ml
	Y1	−	−	−	2,034 EU/ml	1,484 EU/ml	115 EU/ml
	Y2	−	−	−	1,115 EU/ml	837 EU/ml	124 EU/ml
	Y3	−	−	−	899 EU/ml	642 EU/ml	136 EU/ml
	Y4	78/79 (98.7, 93.1–100.0)	52/52 (100.0, 93.2–100.0)	120/120 (100, 97.0–100.0)	748 EU/ml (648–865)	520 EU/ml (422–641)	137 EU/ml (106–178)
Safaeian 2018/CVT LTFU [16]	Y4	165/165 (100.0, 97.8–100.0)	61/61 (100.0, 95.4–100.0)	104/104 (100.0, 96.5–100.0)	803 EU/ml (708–909)	555 EU/ml (447–690)	205 EU/ml (165–255)
	Y7	165/165 (100.0, 97.8–100.0)	61/61 (100.0, 95.4–100.0)	104/104 (100.0, 96.5–100.0)	716 EU/ml (630–814)	460 EU/ml (367–576)	194 EU/ml (158–237)
Kreimer 2020/CVT LTFU [21]	Y9	1,365/1,365 (100.0, 99.7–100.0)	62/62 (100.0, 94.2–100.0)	112/112 (100.0, 96.8–100.0)	699 EU/ml (606–807)	414 EU/ml (328–524)	172 EU/ml (141–209)
	Y11	1,365/1,365 (100.0, 99.7–100.0)	62/62 (100.0, 94.2–100.0)	112/112 (100.0, 96.8–100.0)	664 EU/ml (570–772)	340 EU/ml (267–434)	176 EU/ml (145–214)
Watson-Jones 2022/DoRIS [13]	M0	−	−	−	<LOD	<LOD	<LOD
	M1	−	−	−	50 IU/ml (43–59)	52 IU/ml (46–59)	48 IU/ml (42–56)
	M7	140/141 (99.3, 96.1–100.0)	142/142 (100.0, 97.4–100.0)	147/148 (99.3, 96.3–100.0)	2,658 IU/ml (2,221–3,182)	1,643 IU/ml (1,445–1,868)	16 IU/ml (14–19)
	Y1	141/141 (100.0, 97.4–100.0)	140/140 (100.0, 97.4–100.0)	146/147 (99.3, 96.3–100.0)	641 IU/ml (539–762)	268 IU/ml (232–309)	19 IU/ml (17–23)
	Y2	141/141 (100.0, 97.4–100.0)	141/141 (100.0, 97.4–100.0)	147/148 (99.3, 96.3–100.0)	412 IU/ml (357–475)	163 IU/ml (141–188)	23 IU/ml (20–26)

**HPV18**							
Safaeian 2013/CVT [15]^d^	M0	−	−	−	<LOD	<LOD	<LOD
	M6	−	−	−	408 EU/ml	53 EU/ml	76 EU/ml
	Y1	−	−	−	827 EU/ml	763 EU/ml	71 EU/ml
	Y2	−	−	−	471 EU/ml	446 EU/ml	69 EU/ml
	Y3	−	−	−	369 EU/ml	358 EU/ml	74 EU/ml
	Y4	−	−	−	335 EU/ml (285–392)	305 EU/ml (238–391)	70 EU/ml (54–91)
Safaeian 2018/CVT LTFU [16]	Y4	165/165 (100.0, 97.8–100.0)	61/61 (100.0, 95.4–100.0)	104/104 (100.0, 96.5–100.0)	360 EU/ml (313–414)	296 EU/ml (240–366)	112 EU/ml (93–134)
	Y7	165/165 (100.0, 97.8–100.0)	61/61 (100.0, 95.4–100.0)	104/104 (100.0, 96.5–100.0)	322 EU/ml (281–369)	270 EU/ml (221–330)	125 EU/ml (105–150)
Kreimer 2020/CVT LTFU [21]	Y9	1,365/1,365 (100.0, 99.7–100.0)	62/62 (100.0, 94.2–100.0)	112/112 (100.0, 96.8–100.0)	292 EU/ml (249–342)	210 EU/ml (171–259)	102 EU/ml (83–125)
	Y11	1,365/1,365 (100.0, 99.7–100.0)	62/62 (100.0, 94.2–100.0)	112/112 (100.0, 96.8–100.0)	275 EU/ml (234–323)	194 EU/ml (156–241)	109 EU/ml (89–133)
Watson-Jones 2022/DoRIS [13]	M0	−	−	−	<LOD	<LOD	<LOD
	M1	−	−	−	18 IU/ml (16–21)	18 IU/ml (15–21)	19 IU/ml (16–22)
	M7	135/136 (99.3, 96.0–100.0)	141/141 (100.0, 97.4–100.0)	139/141 (98.6, 95.0–99.8)	727 IU/ml (607–870)	582 IU/ml (505–670)	8 IU/ml (6–9)
	Y1	136/136 (100.0, 97.3–100.0)	139/139 (100.0, 97.4–100.0)	139/140 (99.3, 96.1–100.0)	159 IU/ml (132–190)	96 IU/ml (83–111)	9 IU/ml (7–10)
	Y2	136/136 (100.0, 97.3–100.0)	140/140 (100.0, 97.4–100.0)	139/141 (98.6, 95.0–99.8)	107 IU/ml (90–126)	50 IU/ml (43–58)	10 IU/ml (9–11)

Abbreviations: 2vHPV, bivalent HPV [vaccine]; CI, confidence interval; CVT, Costa Rica Vaccine Trial; EU, ELISA unit; GM, geometric mean; GSK, GlaxoSmithKline; HPV, human papillomavirus; IU, international unit; LOD, limit of detection; LTFU, long-term follow-up; M, month; ml, millilitre; Y, year.

a Definitions of seropositivity used in each study are provided in Supplementary Table 8.

b Seropositivity proportions (%) and 95%CI were calculated by the authors of the systematic review using data provided in the included articles.

c Results are shown only for two-dose arms where participants received dose one at day 0 and dose two at day 180.

d HPV GMCs (95%CI) among 113 unvaccinated but naturally infected controls were 15 (11–19) for HPV16 and 15 (12–19) for HPV18.

**Table 5:** HPV16 and HPV18 seropositivity and geometric mean antibody level results from articles evaluating one versus two or three doses of the 4vHPV or 9vHPV vaccine.

Reference/study	Time point	# seropositive^a^/N participants (% Seropositive, 95 %CI^b^)			GM concentrations/MFI (95 %CI)		
		3 doses	2 doses^c^	1 dose	3 doses	2 doses^c^	1 dose
**Merck 4vHPV^d^**							
**HPV16**							
Sankaranarayanan 2016/IARC India Study [18]	M0	46/1,000 (4.6, 3.4–6.1)	52/937 (5.5, 4.2–7.2)	−	MFI 11 (10–12)	MFI 9 (8–10)	−
	M7	308/308 (100.0, 98.8–100.0)	316/317 (99.7, 98.3–100.0)	−	MFI 5,460 (5,195–5.738)	MFI 6,125 (5,785–6,485)	−
	Y1	−	−	260/528 (49.2, 44.9–53.6)	−	−	MFI 106 (96–116)
	Y1.5	311/313 (99.4, 97.7–99.9)	312/314 (99.4, 97.7–99.9)	255/476 (53.6, 49.0–58.1)	MFI 1,209 (1,105–1,323)	MFI 1,222 (1,116–1,338)	MFI 113 (102–126)
	Y3	225/271 (83.0, 78.0–87.3)	197/278 (70.9, 65.1–76.1)	166/510 (32.5, 28.5–36.8)	MFI 221 (197–247)	MFI 163 (147–181)	MFI 72 (66–78)
Sankaranarayanan 2018/IARC India Study [19]	Y3	271/271 (100.0, 98.6–100.0)	278/278 (100.0, 98.7–100.0)	510/510 (100.0, 99.3–100.0)	MFI 221 (197–247)	MFI 163 (147–181)	MFI 72 (66–78)
	Y4	239/239 (100.0, 98.5–100.0)	243/243 (100.0, 98.5–100.0)	397/397 (100.0, 99.1–100.0)	MFI 196 (170–226)	MFI 197 (172–225)	MFI 86 (75–99)
Joshi 2023/IARC India Study [25]	M7	154/154 (100.0, 97.6–100.0)	−	−	1,045 IU/ml (917–1,191)	−	−
	Y1	−	−	148/150 (98.7, 95.3–99.8)	−	−	10 IU/ml (8–11)
	Y1.5	154/154 (100.0, 97.6–100.0)	−	146/148 (98.6, 95.2–99.8)	129 IU/ml (113–148)	−	8 IU/ml (7–10)
	Y3	136/136 (100.0, 97.3–100.0)	−	147/150 (98.0, 94.3–99.6)	77 IU/ml (66–91)	−	8 IU/ml (7–9)
	Y10	167/167 (100.0, 97.8–100.0)	190/190 (100.0, 98.0–100.0)	311/324 (96.0, 93.2–97.8)	35 IU/ml (30–41)	35 IU/ml (30–40)	10 IU/ml (9–11)

**HPV18**							
Sankaranarayanan 2016/IARC India Study [18]	M0	41/1,000 (4.1, 3.0–5.5)	63/937 (6.7, 5.2–8.5)	−	MFI 6 (5–7)	MFI 5 (4–5)	−
	M7	308/308 (100.0, 98.8–100.0)	317/317 (100.0, 98.8–100.0)	−	MFI 2,942 (2,733–3,167)	MFI 3,068 (2,812–3,347)	−
	Y1	−	−	304/528 (57.6, 53.2–61.8)	−	−	MFI 50 (45–55)
	Y1.5	307/313 (98.1, 85.9–99.3)	305/314 (97.1, 94.6–98.7)	259/476 (54.4, 49.8–59.0)	MFI 377 (337–422)	MFI 269 (241–299)	MFI 46 (40–51)
	Y3	249/271 (91.9, 88.0–94.8)	238/278 (85.6, 80.9–89.5)	271/510 (53.1, 48.7–57.5)	MFI 184 (162–208)	MFI 117 (104–132)	MFI 45 (41–49)
Sankaranarayanan 2018/IARC India Study [19]	Y3	271/271 (100.0, 98.6–100.0)	278/278 (100.0, 98.7–100.0)	510/510 (100.0, 99.3–100.0)	MFI 184 (162–208)	MFI 117 (104–132)	MFI 45 (41–49)
	Y4	239/239 (100.0, 98.5–100.0)	243/243 (100.0, 98.5–100.0)	397/397 (100.0, 99.1–100.0)	MFI 133 (115–154)	MFI 120 (105–136)	MFI 47 (41–53)
Joshi 2023/IARC India Study [25]	M7	154/154 (100.0, 97.6–100.0)	−	−	380 IU/ml (324–445)	−	−
	Y1	−	−	150/150 (100.0, 97.6–100.0)	−	−	3 IU/ml (3–4)
	Y1.5	154/154 (100.0, 97.6–100.0)	−	144/148 (97.3, 93.2–99.3)	33 IU/ml (28–40)	−	3 IU/ml (2–3)
	Y3	136/136 (100.0, 97.3–100.0)	−	147/150 (98.0, 94.2–99.6)	19 IU/ml (15–24)	−	2 IU/ml (2–3)
	Y10	167/167 (100.0, 97.8–100.0)	186/190 (97.9, 94.7–99.4)	314/324 (96.9, 94.4–98.5)	8 IU/ml (7–10)	7 IU/ml (6–8)	3 IU/ml (2–3)

**Merck 9vHPV**							
**HPV16**							
Watson-Jones 2022/DoRIS [13]	M0	−	−	−	<LOD	<LOD	<LOD
	M1	−	−	−	57 IU/ml (50–64)	51 IU/ml (43–59)	55 IU/ml (48–63)
	M7	140/140 (100.0, 97.4–100.0)	142/142 (100.0, 97.4–100.0)	144/144 (100.0, 97.5–100.0)	1,025 IU/ml (896–1,174)	1,401 IU/ml (1,253–1,566)	16 IU/ml (13–19)
	Y1	140/140 (100.0, 97.4–100.0)	142/142 (100.0, 97.4–100.0)	145/145 (100.0, 97.5–100.0)	218 IU/ml (189–251)	253 IU/ml (219–291)	13 IU/ml (12–15)
	Y2	140/140 (100.0, 97.4–100.0)	141/141 (100.0, 97.4–100.0)	144/145 (99.3, 96.2–100.0)	118 IU/ml (102–137)	125 IU/ml (107–146)	14 IU/ml (12–16)

**HPV18**							
Watson-Jones 2022/DoRIS [13]	M0	−	−	−	<LOD	<LOD	<LOD
	M1	−	−	−	19 IU/ml (17–22)	17 IU/ml (15–20)	20 IU/ml (17–23)
	M7	142/142 (100.0, 97.4–100.0)	137/137 (100.0, 97.3–100.0)	133/135 (98.5, 94.8–99.8)	383 IU/ml (334–440)	400 IU/ml (352–454)	7 IU/ml (6–8)
	Y1	142/142 (100.0, 97.4–100.0)	137/137 (100.0, 97.3–100.0)	131/136 (96.3, 91.6–98.8)	67 IU/ml (57–79)	59 IU/ml (50–69)	5 IU/ml (4–6)
	Y2	141/142 (99.3, 96.1–100.0)	136/136 (100.0, 97.3–100.0)	133/136 (97.8, 93.7–99.5)	32 IU/ml (27–38)	29 IU/ml (25–35)	6 IU/ml (5–7)

Abbreviations: 4vHPV, quadrivalent HPV [vaccine]; 9vHPV, nonavalent HPV [vaccine]; CI, confidence interval; GM, geometric mean; HPV, human papillomavirus; IARC, International Agency for Research on Cancer; IU, international unit; LOD, limit of detection; M, month; MFI, median fluorescence intensity; ml, millilitre; Y, year.

a Definitions of seropositivity used in each study are provided in Supplementary Table 8.

b Seropositivity proportions (%) and 95%CI were calculated by the authors of the systematic review using data provided in the included articles.

c Results are shown only for two-dose arms where participants received dose one at day 0 and dose two at day 180.

Across all studies that provided relevant immunogenicity data, rates of seropositivity for antibodies to HPV16 and HPV18 were very high among HPV vaccine recipients, regardless of the number of vaccine doses received. In the DoRIS trial, almost all (≥98 %) 2vHPV and 4vHPV vaccine recipients had detectable antibodies to HPV16 and HPV18 by Y2. At that timepoint, the proportion of 2vHPV recipients who were seropositive for antibodies to HPV16 and HPV18 were marginally lower in the one-dose arm (HPV16: 99.3 %, 95 %CI 96.3–100.0 %; HPV18: 98.6 %, 95 %CI 95.0–99.8 %) compared to the two-dose (HPV16: 100.0 %, 95 %CI 97.4–100.0 %; HPV18: 100.0 %, 95 %CI 97.4–100.0 %) or three-dose arms (HPV16: 100.0 %, 95 %CI 97.4–100.0 %; HPV18: 100.0 %, 95 %CI 97.3–100.0 %) but, in all cases, 95 %CI were overlapping. Results were similar for 9vHPV recipients.

In the CVT LTFU, all 2vHPV vaccine recipients tested positive for antibodies to both HPV16 and HPV18 at Y11, regardless of whether they received one, two or three doses. In the IARC India Study, all participants in the two-dose and three-dose 4vHPV arms (95 %CI 98.0–100.0 % and 97.8–100.0 %, respectively) had antibodies to HPV16 at Y10, compared with 96.0 % (95 %CI 93.2–97.8 %) in the one-dose arm. Results were similar for seropositivity to HPV6, HPV11 and HPV18.

**Fig. 2:**
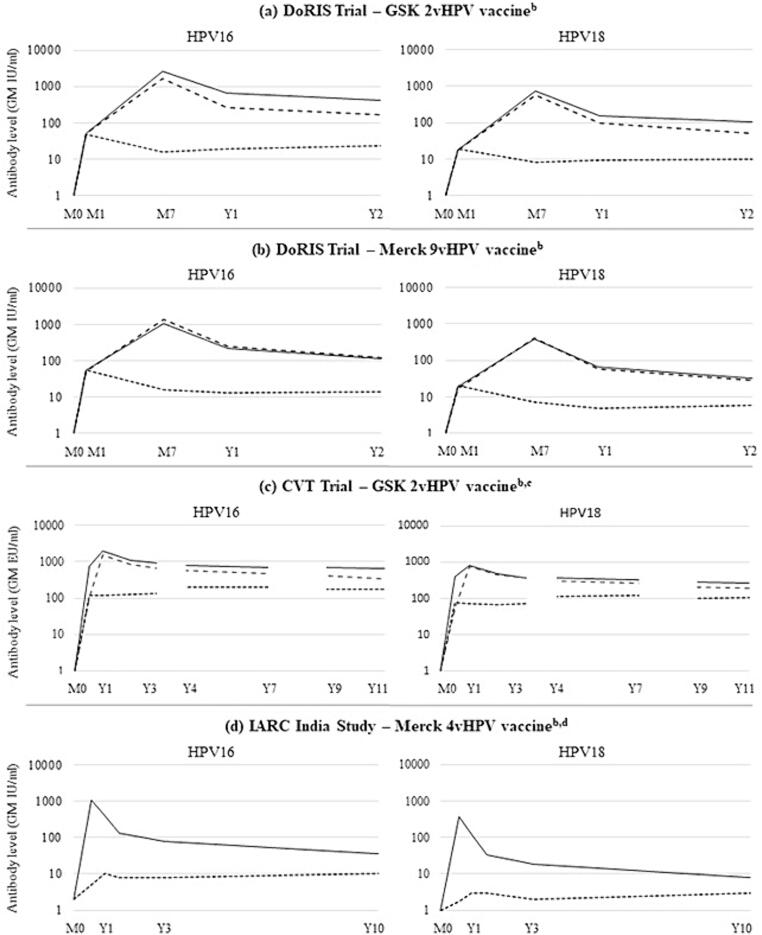
HPV16 and HPV18 geometric mean antibody levels following one dose (dotted line), two doses (dashed line) or three doses (solid line) of HPV vaccine among participants from (a, b) the DoRIS trial, (c) the CVT and (d) the IARC India Study^a^. Abbreviations: 2vHPV, bivalent HPV [vaccine]; 4vHPV, quadrivalent HPV [vaccine]; 9vHPV, nonavalent HPV [vaccine]; CVT, Costa Rica Vaccine Trial; EU, ELISA unit; GM, geometric mean; GSK, GlaxoSmithKline; HPV, human papillomavirus; IARC, International Agency for Research on Cancer; IU, international unit; M, month; ml, millilitre; Y, year.^. a^Graphs were created by the authors of this review using data on GM antibody levels reported in the included articles for each of the studies. ^b^In the DoRIS trial and the IARC India Study, antibody levels were measured in IU/ml. In the CVT, they were measured in EU/ml. Methods used in each study are presented in Supplementary Table 8. ^c^Data presented for CVT participants are derived from three articles: M0 to Y3 data are from Safaeian 2013 [15], Y4-7 data are from Safaeian 2018 [16] and Y9-11 data are from Kreimer 2020 [21]. Antibody levels for the three articles were measured at different points in time (in different batches) and among different (but overlapping) sets of CVT participants. Thus, caution should be taken when interpreting antibody levels over time. ^d^Data are presented from the most recent article from the IARC India Study: Joshi 2023 [25]. Earlier articles from this study reported antibody levels in MFI. The most recent article measured antibody levels in the two-dose arm only at the Y10 timepoint. Thus, levels over time are just presented for the one-dose and three-dose arms.

In all three studies, GMCs were significantly higher following two-dose or three-dose HPV vaccination compared to one-dose vaccination. However, whilst GMCs in the two-dose and the three-dose arms typically reached a peak soon after the last vaccine dose and then declined substantially, reaching a plateau by around two years, those in the one-dose arms typically reached a peak soon after vaccination and then remained stable throughout follow-up (Fig. 2).

In the small US pilot study (which enrolled women who were HPV16 seropositive at baseline), four of the five women vaccinated with one dose of the 4vHPV vaccine (versus none of the unvaccinated women) had increased HPV16 antibody GMCs by month (M)1.

#### Antibody avidity

Five articles present data on HPV16 and/or HPV18 antibody avidity following one dose of HPV vaccine versus a comparator schedule; two from the CVT or CVT LTFU [16], [24], two from the IARC India Study [18], [19]; and one from the DoRIS trial [13]. One article from the IARC India Study also presents data on HPV6 and HPV11 antibody avidity [18].

At Y2 in the DoRIS trial, there was no difference in HPV16 or HPV18 antibody avidity between dose groups for the 2vHPV or 9vHPV vaccine. By Y11 in the CVT, HPV16 antibody avidity was lower in the one-dose 2vHPV arm (2.7, 95 %CI 2.6–2.8) compared to the three-dose arm (3.0, 95 %CI 2.9–3.1), but the difference was small. Conversely, at Y1.5 in the IARC India Study, HPV16 antibody avidity (for MFI) was slightly higher in the one-dose 4vHPV arm (HPV16: 74, 95 %CI 68–80) compared to the two-dose (HPV16: 66, 95 %CI 63–70) and three-dose arms (HPV16: 67, 95 %CI 64–71), though 95 %CI were overlapping. Results were similar for HPV18 avidity, but no differences were observed between groups for HPV6 and HPV11 avidity.

## Discussion

Findings from this systematic review support the notion that one HPV vaccine dose is highly effective, and possibly as effective as two or three doses, at preventing cervical vaccine-type HPV infection and thus cervical cancer in healthy young females (at least to a decade post-vaccination). It thus supports WHO’s recent change in recommendations that allow for either one-dose or two-dose HPV vaccination in girls and women up to 20 years of age [8]. Offering a single-dose HPV vaccine schedule would substantially reduce the costs and simplify the logistics of HPV vaccine delivery, freeing up healthcare staff and resources for strengthening one-dose delivery and coverage, as well as other adolescent health services. Furthermore, it would increase availability of HPV vaccine doses for countries that have not yet been able to introduce HPV vaccination into their national immunisation schedules, and potentially enable access for other important target groups who are currently not prioritised for HPV vaccination in most countries (e.g. older women, males and people with HIV).

Most of the included efficacy studies examined cervical HPV infection as an endpoint. The KEN-SHE trial was the only efficacy study identified that prospectively randomised participants to receive one HPV vaccine dose versus a control comparator (one dose of meningococcal vaccine) [12]. In that study, single-dose efficacy was extremely high, albeit with follow-up only to 1.5 years post-vaccination (though recent data to M36 of follow-up presented at the 2023 International Papillomavirus Conference gave the same conclusions [27]). Whilst a multidose comparator group was not included, one dose offered near complete protection for vaccine-related genotypes, providing little or no room for improvement with additional doses in the short term.

Accordingly, observational data from the earlier trials provide evidence that one HPV vaccine dose is as efficacious as two or three doses [14], [16], [17], [18], [19], [21], [22], [23], [24], with the CVT and IARC India data now extending to a decade or more post-vaccination [21], [22], [23]. However, the relatively small numbers of participants contributing to the reduced dose efficacy data from these studies, and thus the limited power to detect differences across dose groups, should be noted.

The IARC India Study is the only study identified that examined CIN/ICC outcomes. By 10 years post-vaccination, HPV16/18-associated CIN events were detected in almost 10-times the number of unvaccinated compared to HPV-vaccinated participants (with no events in the one-dose arm), although the actual numbers of CIN cases are very small at this stage in the study [23]. Follow-up of women for ongoing accrual of cervical disease outcomes will continue to ≥15 years post-vaccination.

Whilst several non-trial observational studies conducted to date have examined the effectiveness of one-dose HPV vaccination against non-cervical HPV infection [10], we identified only one such study (investigating oral infections) from a trial setting. The study provided no evidence of a difference in the prevalence of vaccine-type oral infections between dose groups, though it was under-powered for this comparison [26]. The study authors concluded that one HPV vaccine dose may be less effective than two or three doses in preventing oral HPV infection, though this was not supported by the published data. Whilst prevalence point estimates for a couple of HPV types/combinations (notably including non-vaccine-type HPV infections) differed across groups, 95 %CI were wide and overlapping. It is not clear how participants were selected for inclusion in the study so the potential for selection bias is difficult to assess.

Across the studies that examined immunogenicity endpoints, most HPV-vaccinated participants produced detectable antibody responses to HPV16 and HPV18, though different studies used different antibody detection assays and cut-off criteria. Using the methods applied in this systematic review, there was little or no evidence for a difference in HPV16 or HPV18 seropositivity across dose groups in most studies [13], [15], [16], [19], [21], [25]. However, in the DoRIS trial seropositivity analyses, the investigators specifically assessed for non-inferiority, whereby a one-dose schedule was considered to be non-inferior to a two-dose or three-dose schedule if seropositivity was reduced by no more than 5 % [13]. At M24, non-inferiority criteria were met for both vaccines evaluated for HPV16, but not for HPV18 (even though ≥98 % of single-dose participants had HPV18 antibodies).

As expected, across all immunogenicity studies, HPV16 and HPV18 antibody levels were substantially lower with one HPV vaccine dose compared to two or three doses. However, within the one-dose groups, antibody levels reached a plateau soon after vaccination and were then remarkably stable over time [21], [23], [28], showing no evidence of a decline by a decade post-vaccination [21], [23]; and antibody avidity (a measure of the strength of antibody binding) was similar across dose groups [13], [16], [18], [24]. Currently, there is no known correlate of protection against HPV infection. However, despite having lower antibody levels, one-dose recipients from the CVT and the IARC India Study were found to have similarly low incidences of vaccine-type HPV infection as multidose participants, indicating that the antibody levels observed with one dose are sufficient for protection [16], [21]. In separate immunobridging studies (not eligible for inclusion in this systematic review), the HPV16 and HPV18 antibody levels observed among one-dose recipients in the DoRIS trial were non-inferior to those observed among one-dose recipients in the CVT, the IARC India Study and the KEN-SHE trial [29], [30]. Given that one dose was demonstrated to be efficacious in those three studies, the immunobridging results indicate that the antibody levels observed with one dose in the DoRIS trial are highly likely to be sufficient for protection.

Our systematic review was limited by the small number of prospectively designed trials of single-dose HPV vaccination available for inclusion and, whilst the observational evidence extends to a decade post-vaccination, the data from prospective trials currently extend only to two years post-vaccination (though key conclusions did not change for either trial for M36 data presented at recent scientific conferences [27], [28]). One of the few intervention studies included in our review provided very little relevant data [20]. The primary aim of that study was to examine memory B cell responses, which was outside the scope of our review; and only minimal antibody data were presented. Furthermore, all studies eligible for inclusion to date have been conducted among healthy young females and used the GSK 2vHPV vaccine or the Merck 4vHPV or 9vHPV vaccine. Evidence gaps thus remain for other important populations such as girls and women with HIV and males, and for new HPV vaccines recently licensed for use in some countries [8].

Recent data from several clinical trials (albeit ineligible for inclusion in our review due to publication after our most recent search date [31], no data from an unvaccinated or multidose HPV vaccine comparator group presented at the same time point as for the single-dose group [31], [32], or insufficient data on one-dose HPV vaccination [33]) provide some limited evidence that one dose of the 9vHPV vaccine is immunogenic in adolescent boys [31], and that one dose of the new 2vHPV vaccine (Cecolin®) manufactured by Xiamen Innovax Biotech Co. Ltd. may be immunogenic and efficacious against HPV16/18 infection [32], [33], but further studies are needed.

Importantly, several ongoing trials and studies evaluating the efficacy, immunogenicity and/or population-level impact of single-dose HPV vaccination will add to the evidence-base in healthy young girls and women and address some of the key evidence gaps over the next few years [9]. These include the longer-term follow-up of the DoRIS trial, the CVT and the IARC India study, and other studies from Tanzania [34], Costa Rica [35], [36], [37], Thailand [38], South Africa [39], the Gambia [40], and Brazil, Haiti and Peru [41]. In addition, the National Technical Advisory Group on Immunization (NTAGI) in India met in July 2023 to discuss the Serum Institute of India’s quadrivalent HPV vaccine and proposed a cohort study of girls who receive a single dose, as well as follow-up of girls who missed a second dose, to generate data on the immunogenicity and effectiveness of one-dose HPV vaccination [42].

Large, prospectively designed studies that overcome many of the limitations of the earlier non-randomised studies will be crucial for confirming efficacy of single-dose vaccination. Even if individual-level efficacy is found to be lower with one dose than with two or three doses, the population-level impact of a single-dose strategy could be substantial, particularly if it allows more widespread HPV vaccine introduction and higher vaccine coverage.

Based on the strength of the data available to date and following the change in WHO recommendations for HPV vaccine dosing schedules, many countries (including England, Australia, India, Bangladesh, Nigeria and Tanzania, among others) have chosen or are considering to switch from a two-dose to one-dose schedule in healthy adolescents, or to introduce the vaccine as a single dose schedule [43]. Mathematical modelling indicates that switching to a one-dose schedule would not substantially increase cervical cancer cases if one dose provides ≥20 years of protection [44]; if ongoing clinical trials unexpectedly indicate a potentially concerning shorter duration of protection, there will be sufficient time to revert to a two-dose schedule before any impact is seen in cervical cancer rates [45]. Nonetheless, some countries have opted to wait until availability of upcoming clinical trial evidence prior to deciding to switch from a multidose to one-dose schedule. However, for many countries that do not yet offer HPV vaccination within national immunisation schedules (mostly LMICs, where the greatest cervical cancer burden occurs [5]), the decision that must be made is whether to introduce one-dose or two-dose vaccination now, or whether to wait to introduce any vaccine. For those countries, modelling shows that the most detrimental scenario is to wait [46].

## Author contributions

HK, HSW and KEG developed the review protocol, and HSW developed the search strategy with input from HK, KEG and DWJ. HSW performed database searches, and HSW, KEG, NH, SMJ, GM and EMC double-screened search results against eligibility criteria. HSW performed data extraction and analyses, conducted the narrative synthesis, and produced data tables, which were verified by HK and EMC. KEG and HSW conducted the quality assessment. All authors reviewed and agreed upon the review results. HSW wrote the manuscript with input and revision by all authors. All authors approved the version for submission, and all authors attest they meet the ICMJE criteria for authorship.

## Disclaimer

Where authors are identified as personnel of the International Agency for Research on Cancer/World Health Organization, the authors alone are responsible for the views expressed in this article and they do not necessarily represent the decisions, policy or views or the International Agency for Research on Cancer/World Health Organization.

## Funding

This work was supported, in whole or in part, by the 10.13039/100000865Bill & Melinda Gates Foundation [INV-008475]. Under the grant conditions of the Foundation, a Creative Commons Attribution 4.0 Generic License has already been assigned to the Author Accepted Manuscript version that might arise from this submission.

SMJ is funded by the 10.13039/501100000272National Institute for Health and Care Research (NIHR) Health Protection Research Unit in Vaccines and Immunisation (NIHR200929). NH is funded by the National University of Singapore Saw Swee Hock School of Public Health.

## CRediT authorship contribution statement

**Hilary S. Whitworth:** Writing – review & editing, Writing – original draft, Validation, Supervision, Methodology, Investigation, Formal analysis, Data curation, Conceptualization. **Sandra Mounier-Jack:** Writing – review & editing, Investigation, Data curation. **Edward M. Choi:** Writing – review & editing, Validation, Investigation, Data curation. **Katherine E. Gallagher:** Writing – review & editing, Methodology, Investigation, Data curation, Conceptualization. **Natasha Howard:** Writing – review & editing, Investigation, Data curation. **Helen Kelly:** Writing – review & editing, Validation, Methodology, Investigation, Conceptualization. **Gladys Mbwanji:** Writing – review & editing, Investigation, Data curation. **Aimée R Kreimer:** Writing – review & editing, Investigation. **Partha Basu:** Writing – review & editing, Investigation. **Ruanne Barnabas:** Writing – review & editing, Investigation. **Mélanie Drolet:** Writing – review & editing, Investigation. **Marc Brisson:** Writing – review & editing, Investigation. **Deborah Watson-Jones:** Writing – review & editing, Supervision, Project administration, Methodology, Investigation, Funding acquisition, Conceptualization.

## Declaration of competing interest

The authors declare the following financial interests/personal relationships which may be considered as potential competing interests: DWJ has received research funding and HPV vaccine donations from MSD and GlaxoSmithKline Biologicals. Regeneron Pharmaceuticals covered RVB’s cost of conference abstract and manuscript writing, outside the submitted work. RVB serves on a Gilead Sciences DMC, for which she is paid an honorarium. HSW, ARK, PB, RB and DWJ are investigators in ongoing studies and clinical trials evaluating the efficacy and/or immunogenicity of single- dose HPV vaccination: the IARC India vaccine trial (PB), CVT (ARK), the DoRIS trial (DWJ, HSW), the ESCUDDO trial (ARK), the Primavera trial (ARK), the KEN-SHE trial (RVB) and the Add-Vacc trial (DWJ, HSW). HSW, SMJ, ARK, PB, RVB, MD, MB and DWJ are members of the PATH-convened Single-Dose HPV Vaccine Evaluation Consortium. SMJ is funded by the National Institute for Health and Care Research (NIHR) Health Protection Research Unit in Vaccines and Immunization (NIHR200929).

## Data Availability

Data will be made available on request.
