# Supplementary material for: Efficacy and immunogenicity of a single dose of human papillomavirus vaccine compared to multidose vaccination regimens or no vaccination: An updated systematic review of evidence from clinical trials
Source: Vaccine X. 2024 Apr 16;19:100486. doi: 10.1016/j.jvacx.2024.100486 (PMC11169951; doi:10.1016/j.jvacx.2024.100486)
Supplement: Supplementary Data 1 [file mmc1.docx]

**SUPPLEMENTARY MATERIAL**

**for the manuscript**

**‘Efficacy and immunogenicity of a single dose of Human Papillomavirus vaccine compared to multidose vaccination regimens or no vaccination: An updated systematic review of evidence from clinical trials’**

**SUPPLEMENTARY RESULTS**

**Intervention studies of one-dose HPV vaccination included in the systematic review**

*The KEN-SHE trial*

One article in this review presents results from the KEN-SHE trial, which is an ongoing double-blind randomised controlled trial (RCT) evaluating the efficacy and immunogenicity of one dose of the 2vHPV vaccine or the 9vHPV vaccine among 2,275 healthy, sexually active girls and women aged 15-20 years in Kenya (ClinicalTrials.gov: NCT03675256) (1). Girls and women were randomised to receive one dose of either of the two HPV vaccines or a control (meningococcal) vaccine at month (M)0, and were followed up to year (Y)3 for efficacy (against HPV infection) and immunogenicity assessments (2). The primary analysis visit was Y1.5, and the relevant article included in this review presents efficacy data up to that timepoint (1).

*The DoRIS trial*

One article in our review presents results from the DoRIS trial, which is an ongoing open-label RCT comparing the immunogenicity of one versus two versus three doses of the 2vHPV vaccine or the 9vHPV vaccine in 930 healthy, HIV-negative Tanzanian schoolgirls aged 9-14 years (ClinicalTrials.gov: NCT02834637) (3). Girls were allocated to one of the six vaccine schedules at M0 and were initially followed up to Y3 for immunogenicity assessments (4). Girls in the one-dose and two-dose arms are now continuing in follow-up to Y9. The relevant article included in this systematic review presents immunogenicity results from the DoRIS trial up to Y2, the primary outcome visit (3).

*The US pilot study*

One article in our review presents data from a small pilot study, which was conducted in the United States (US) to evaluate memory B cell responses following one dose of the 4vHPV vaccine in healthy women aged 27-45 years with prior HPV16 infection (5). Ten HPV16-seropositve women were randomised at M0 to receive one HPV vaccine dose or no vaccination and were followed up to M6 to examine antibody and memory B cell responses.

**Observational studies of one-dose HPV vaccination included in the systematic review**

*CVT*

Seven of the articles included in our review present data from the Costa Rica Vaccine Trial (CVT), or from the long-term follow-up (LTFU) of CVT participants (6-12). The CVT was a double-blind RCT that evaluated the efficacy and immunogenicity of a three-dose schedule of the 2vHPV vaccine among healthy Costa Rican women aged 18-25 years (ClinicalTrials.gov: NCT00128661) (13). At M0, 7,466 women were randomised to receive three doses of HPV vaccine or three doses of a control (hepatitis A [HAV]) vaccine. The women were initially followed up to Y4 for efficacy (against HPV infection) and immunogenicity assessments. At that point, women in the HPV vaccine arm were enrolled in a LTFU study, which is currently continuing follow-up to Y20. At Y4, women in the CVT control arm were offered HPV vaccination, and thus a separate control group of age-matched healthy unvaccinated females was recruited for the LTFU study.

In the CVT, some women did not complete their allocated vaccine schedule (instead receiving one or two HPV vaccine or HAV vaccine doses), primarily due to pregnancy, colposcopy referral, other medical conditions, vaccine refusal or missed study visits. Four of the articles included in this review present efficacy and/or immunogenicity data from CVT participants who received one versus two versus three vaccine doses: two up to Y4 (6, 7), one up to Y7 (8) and one up to Y11 (9). A separate article presents combined Y4 efficacy data from the CVT and the PATRICIA trial (described below) (12). One further article describes an evaluation of cross-protection of the different vaccine schedules against non-vaccine-type HPV infections up to Y11 (10), and another describes an evaluation of HPV16 avidity patterns over the same time period among one-dose and three-dose recipients (11).

*The PATRICIA trial*

The PATRICIA trial was a large multi-country double-blind RCT that evaluated the efficacy of a three-dose schedule of the 2vHPV vaccine among 18,729 healthy women aged 15-25 years (ClinicalTrials.gov: NCT001226810) (14). The study design was similar to that of CVT; women were randomised to receive three doses of HPV vaccine or HAV vaccine and were followed up to Y4 for efficacy (against HPV infection) and immunogenicity assessments. As in the CVT (and for similar reasons), some women did not receive all their allocated vaccine doses, instead receiving just one or two doses. As described above, the combined CVT/PATRICIA article that was included in our systematic review presents efficacy data up to Y4 (12).

*The IARC India Vaccine Study*

Four of the articles included in our review present data from the IARC India Study (15-18), and a further article presents data from a nested sub-study (19). The IARC India Study was originally intended to be an open-label cluster-randomised trial comparing two versus three doses of the 4vHPV vaccine among healthy unmarried girls aged 10-18 years from 188 geographical clusters in India (ClinicalTrials.gov: NCT00923702) (15). However, early in the trial, the Indian government suspended all HPV vaccine trials (for reasons unrelated to the study), resulting in early termination of enrolment and vaccinations. In total, 17,729 girls (almost 90% of the target number) were enrolled in the study, but many of these did not receive their full allocated vaccine schedule, with some receiving just one dose. Girls commenced follow-up for efficacy (against HPV infection and cervical intraepithelial neoplasia [CIN] / invasive cervical cancer [ICC]) and immunogenicity assessments nonetheless; but, due to the disruption in randomisation, the originally planned clinical trial of two versus three HPV vaccine doses became a prospective observational cohort study of one versus two versus three vaccine doses. Follow-up is ongoing and planned to ≥Y15. Two years into the study, a control group of age-matched married, unvaccinated females was enrolled for comparison. Approximately 8-10 years into the study, a second control group of age-matched married, unvaccinated females was enrolled specifically for comparison in cervical cancer screening (for CIN and ICC outcomes).

Four of the relevant articles included in this review present efficacy and/or immunogenicity data from girls who received one versus two versus three vaccine doses: one up to Y4 (15), one up to Y7 (16) and two up to Y10 (17, 18). The fifth article describes a sub-study that evaluated the impact of one versus two versus three HPV vaccine doses on oral HPV infections (19).

**SUPPLEMENTARY TABLES**

**Supplementary Table 1:** Search terms and limits used, by database.

**Supplementary Table 2:** Relevant review articles identified through database searches and used for reference list screening.

**Supplementary Table 3:** Study eligibility criteria, conforming to PICOS format.

**Supplementary Table 4:** Quality assessment of included studies.

**Supplementary Table 5:** Sampling, laboratory methods and analyses performed and reported by each study/article for infection outcome measures.

**Supplementary Table 6:** Vaccine-type infection results from articles comparing one dose of the 4vHPV or 9vHPV vaccine to either no HPV vaccination or multidose schedules.

**Supplementary Table 7:** HPV31/33/45 infection results from articles comparing one dose of the 2vHPV vaccine or the 4vHPV vaccine to either no HPV vaccination or multidose schedules.

**Supplementary Table 8:** Sampling, laboratory methods and analyses performed and reported by each study/article for immunogenicity outcome measures.

**Supplementary Table 9:** HPV6 and HPV11 seropositivity and geometric mean antibody level results from articles evaluating one versus two or three doses of the 4vHPV vaccine.

**Supplementary Table 10:** HPV antibody avidity results from articles that evaluated one versus two or three doses of HPV vaccine.

**Supplementary Table 1:** Search terms and limits used, by database.

| **Database** | **Search terms^a^** | **Limits** |
| --- | --- | --- |
| MEDLINE | ((HPV* OR papillomavirus* OR papilloma-virus*) OR (exp Papillomavirus Infections/)) AND ((Vaccin* OR Immunis* OR Immuniz* OR Cervarix OR Gardasil OR Valent OR Bivalent OR Quadrivalent OR Cecolin) OR (exp Papillomavirus Vaccines/)) AND ((Immuno* OR Immunit* OR Immune* OR Humoral OR Serolog* OR Antibod* OR Level* OR Titer* OR Avidit*) OR (exp Immune System Phenomena/) OR (Infection* OR Prevalen* OR Incidence OR Efficac* OR Effectiveness OR Cancer* OR Precancer* OR Tumour* OR Tumor* OR Oncol* OR Malignan* OR Lesion* OR Neoplasia* OR Dysplasia* OR Hyperplasia* OR Wart* OR Mouth Papilloma* OR Papillomatosis) OR (exp "Pathological Conditions, Signs and Symptoms"/) OR (exp Epidemiologic Methods/)) AND ((Dose* OR Dosing OR Dosage* OR Schedule* OR Complet* OR Uptake OR Coverage) OR (exp Immunization Schedule/)) | Humans only, Date range applied |
| EMBASE | ((HPV* OR papillomavirus* OR papilloma-virus*) OR (exp Papillomavirus Infection/)) AND ((Vaccin* OR Immunis* OR Immuniz* OR Cervarix OR Gardasil OR Valent OR Bivalent OR Quadrivalent OR Cecolin) OR (Wart Virus Vaccine/)) AND ((Immuno* OR Immunit* OR Immune* OR Humoral OR Serolog* OR Antibod* OR Level* OR Titer* OR Avidit*) OR (exp Immunogenicity/) OR (Infection* OR Prevalen* OR Incidence OR Efficac* OR Effectiveness OR Cancer* OR Precancer* OR Tumour* OR Tumor* OR Oncol* OR Malignan* OR Lesion* OR Neoplasia* OR Dysplasia* OR Hyperplasia* OR Wart* OR Mouth Papilloma* OR Papillomatosis) OR (exp neoplasm/ OR exp verruca vulgaris/ OR exp Epidemiological Data/)) AND ((Dose* OR Dosing OR Dosage* OR Schedule* OR Complet* OR Uptake OR Coverage) OR (exp dosage schedule comparison/)) | Humans only, Date range applied |
| Global Health Database | ((HPV* OR papillomavirus* OR papilloma-virus*) OR (exp Human Papillomaviruses/)) AND ((Vaccin* OR Immunis* OR Immuniz* OR Cervarix OR Gardasil OR Valent OR Bivalent OR Quadrivalent OR Cecolin) OR (exp vaccination/)) AND ((Immuno* OR Immunit* OR Immune* OR Humoral OR Serolog* OR Antibod* OR Level* OR Titer* OR Avidit*) OR (exp Immune Response/) OR (Infection* OR Prevalen* OR Incidence OR Efficac* OR Effectiveness OR Cancer* OR Precancer* OR Tumour* OR Tumor* OR Oncol* OR Malignan* OR Lesion* OR Neoplasia* OR Dysplasia* OR Hyperplasia* OR Wart* OR Mouth Papilloma* OR Papillomatosis) OR (exp Neoplasms/ OR exp Warts/ OR exp Disease Prevalence/ OR exp Incidence/ OR exp Infection/)) AND ((Dose* OR Dosing OR Dosage* OR Schedule* OR Complet* OR Uptake OR Coverage) OR (exp dosage/)) | Date range applied |
| Cochrane Central Register of Controlled Trials | ((HPV* OR papillomavirus* OR papilloma-virus*) OR (MeSH descriptor: [Papillomavirus Infections] explode all trees)) AND ((Vaccin* OR Immunis* OR Immuniz* OR Cervarix OR Gardasil OR Valent OR Bivalent OR Quadrivalent OR Cecolin) OR (MeSH descriptor: [Papillomavirus Vaccines] explode all trees)) AND ((Immuno* OR Immunit* OR Immune* OR Humoral OR Serolog* OR Antibod* OR Level* OR Titer* OR Avidit*) OR (MeSH descriptor: [Immune System Phenomena] explode all trees) OR (Infection* OR Prevalen* OR Incidence OR Efficac* OR Effectiveness OR Cancer* OR Precancer* OR Tumour* OR Tumor* OR Oncol* OR Malignan* OR Lesion* OR Neoplasia* OR Dysplasia* OR Hyperplasia* OR Wart* OR Mouth Papilloma* OR Papillomatosis) OR (MeSH descriptor: [Pathological Conditions, Signs and Symptoms] explode all trees) OR (MeSH descriptor: [Epidemiologic Methods] explode all trees)) AND ((Dose* OR Dosing OR Dosage* OR Schedule* OR Complet* OR Uptake OR Coverage) OR (MeSH descriptor: [Immunization Schedule] explode all trees)) | Date range applied |

Abbreviations: HPV: Human papillomavirus; MeSH: Medical Subject Headings.

^a^The same search strategy was used for each of the database searches (i.e. in 2018, 2021, 2022 and 2023). The only change was that ‘Cecolin’ was added to the list of search terms after the initial search because the Innovax Cecolin® 2vHPV vaccine received WHO prequalification in 2021.

**Supplementary Table 2:** Relevant review articles identified through database searches and used for reference list screening.

| **Search 1: 01Jan99-14Aug18** |
| --- |
| Stanley M. Alternative dosage schedules with HPV virus-like particle vaccines. Expert Rev Vaccines 2014; 18(12):1309-1316. |
| Toh ZQ et al. Reduced dose human papillomavirus vaccination: An update of the current state-of-the-art. Vaccine 2015;33(39):5042–50. |
| Kreimer AR et al. The case for conducting a randomized clinical trial to assess the efficacy of a single dose of prophylactic HPV vaccines among adolescents. J Natl Cancer Int 2015; 107(3):dju436. |
| Basu P et al. Less than 3 doses of the HPV vaccine – Review of efficacy against virological and disease endpoints. Hum Vacc Immunother. 2016;12(6):1394–402. |
| Markowitz LE et al. Human papillomavirus vaccine effectiveness by number of doses: Systematic review of data from national immunization programs. Vaccine 2018;36(32):4806–15. |
| Stanley M et al. HPV single-dose vaccination: Impact potential, evidence base and further evaluation. Vaccine 2018; 36(32 Pt A):4759-4760. |
| Sampson JN et al. Design and statistical considerations for studies evaluating the efficacy of a single dose of the human papillomavirus (HPV) vaccine. Contemporary Clinical Trials 2018;68:35-44. |
| Kreimer AR et al. Evidence for single-dose protection by the bivalent HPV vaccine-Review of the Costa Rica HPV vaccine trial and future research studies. Vaccine 2018;36(32):4774–82. |
| **Search 2: 01Jan18-26Jul21** |
| Franceschi S et al. Options for design of real-world impact studies of single-dose vaccine schedules. Vaccine. 2018;36(32 Pt A):4816-4822. |
| Brotherton JML et al. More evidence suggesting that 1-dose human papillomavirus vaccination may be effective. Cancer. 2020;126(8):1602-1604. |
| Kanatas A. Can a single dose of the human papilloma virus (HPV) vaccine prevent oropharyngeal cancer? Br J Oral Maxillofac Surg. 2020;58(10):e234-e236. |
| Pal M et al. Single-dose nonavalent HPV vaccine: Need of the hour. Nepal J Epidemiol. 2020; 10(2):871-873. |
| Secor AM et al. Immunogenicity of alternative dosing schedules for HPV vaccines among adolescent girls and young women: A systematic review and meta-analysis. Vaccines. 2020;8(4):1-13. |
| Gallant D et al. Single-Dose Human Papillomavirus Vaccination in Low- and Middle-Income Countries-Time for Implementation? Journal of Pediatric and Adolescent Gynecology. 2021;34(5):586-590. |
| **Search 3: 01Jan21-04Feb22** |
| Shao S et al. A scalable manufacturing approach to single dose vaccination against HPV. Vaccines. 2021;9(1): 1-15. |
| **Search 4: 01Jan22-02Feb23** |
| D'Souza J et al. Ethical considerations for the modification of routine human papillomavirus immunisation schedules. Lancet Oncology. 2022;23(9):1121-1122. |
| Velicer C et al. Using observational data to explore the hypothesis that a single dose of current HPV vaccines can provide durable protection. Vaccine. 2022;40(24):3275-3277. |
| Quang, C et al. Single-dose HPV vaccine immunity: is there a role for non-neutralizing antibodies? Trends in Immunology. 2022;43(10):815-825. |
| Shadab R et al. Key ethical considerations to guide the adjudication of a single-dose HPV vaccine schedule. Human vaccines & Immunotherapeutics. 2022;18(1):1917231. |
| Markowitz LE et al. Human papillomavirus vaccine effectiveness by number of doses: Updated systematic review of data from national immunization programs. Vaccine. 2022;40(37):5413-5432. |
| Kreimer AR et al. Public health opportunities resulting from sufficient HPV vaccine supply and a single-dose vaccination schedule. J Natl Cancer Int. 2023;115(3):246-249. |
| Villa LL et al. HPV vaccination programs in LMIC: is it time to optimize schedules and recommendations? Jornal de Pediatria. 2023;99(Suppl 1):S57-S61. |

**Supplementary Table 3:** Study eligibility criteria, conforming to PICOS format.

| Population or participants | Any population, including females and/or males of any age group, with or without HIV infection. |
| --- | --- |
| Intervention or exposure | One-dose prophylactic HPV vaccine schedule. |
| Comparison groups | 1) Two or three dose HPV vaccine schedule (in any schedule, including but not limited to: 0, 1 months; 0, 2 months; 0, 6 months; 0, 12 months; 0, 1, 6-12 months, 0, 2, 6-12 months); and/or  2) No HPV vaccination. |
| Primary outcomes | Efficacy against:   - Vaccine-type HPV infection (point prevalence, incidence and/or persistence, as well as cumulative measures) - HPV-associated clinical outcomes (including, but not limited to, anogenital warts, cervical intraepithelial neoplasia, squamous intraepithelial lesions)   Humoral immunogenicity against vaccine-type HPV, using the following measures:   - Percentage seroconversion - Antibody level / concentration (neutralising and/or non-neutralising), including GMC or MFI - Antibody avidity index - Antibody stability over time - Antibody avidity over time |
| Secondary outcomes | Efficacy against non-vaccine type HPV infection (prevalence, incidence and/or persistence) and/or non-vaccine type HPV-associated clinical outcomes (stratified by HPV types with known cross-protection, i.e. HPV 31/33/52 & 45, and ‘other HPV types’).  Efficacy against HPV infection (prevalence, incidence and/or persistence) and/or HPV-associated clinical outcomes whereby the HPV type is unknown or not stated.  Other humoral immunogenicity outcomes, as reported by the study. |
| Setting | Any setting within any geographical location. |
| Study designs | Any study including participants who received HPV vaccine through a clinical trial, and where data were presented by number of doses received. This could include the original clinical trial in which vaccine was administered or subsequent/nested observational studies, e.g. prospective cohort or cross-sectional studies. |
| Language | No language restrictions were applied. |
| Exclusion criteria | Studies not meeting inclusion criteria.  Studies of the following: Programme evaluation, National immunisation programmes, Population surveillance, Sentinel surveillance.  Studies that were not conducted in humans.  Studies not published between the specified search dates. |

Abbreviations: GMC: geometric mean concentration; HIV: Human immunodeficiency virus; HPV: Human papillomavirus; MFI: Mean fluorescent intensity.

**Supplementary Table 4:** Quality assessment of included studies.

| **Parameter** | **Summary** |
| --- | --- |
| **CVT, CVT LTFU and PATRICIA trial (6-12)** | |
| Selection bias | CVT and PATRICIA were individually randomized trials of 3d HPV vaccination compared to control HAV. Participants were blinded to vaccine allocation. The 1d HPV vaccine group were non-completers of the 3d schedule (due to pregnancy, referral to colposcopy, medical conditions, refusal of subsequent vaccinations or missed study visits). Confounding factors could differentially affect whether a participant completed the schedule and her risk of HPV infection during follow-up (e.g., pregnancy and colposcopy may indicate higher levels of sexual activity and greater exposure to HPV). However, the prevalence of chlamydia infection, pregnancy and colposcopy were balanced between the HPV 1d group and the HAV 1d control group, against which 1d HPV efficacy was estimated. Analyses also assessed whether groups were comparable with respect to sexual activity by looking at HPV DNA or antibody positivity at enrolment. The 1d group had slightly higher HPV DNA detection at enrolment but similar rates of HPV seropositivity as the 3d group (i.e., the 1d group may have been more sexually active on average, and in theory, this would lead to lower VE in the 1d group), yet the data appear to suggest very high VE in the 1d group despite these differences at baseline. |
| Retention/survival bias | Kreimer et al. 2011 set the primary endpoint as newly detected HPV16/18 at the 6m visit or later. The 6m visit was the time of 3rd vaccine administration, so it is likely that those who missed their 3rd dose in the 1d or 2d groups missed this study visit and therefore had a lower probability of detection of incident HPV detection than the 3d group. However, the VE calculated for the 1d group may still be unbiased as it was calculated against a subset of the HAV control group that attended/missed the same study visits. The later analysis of the same data, combined with the PATRICIA trial data (Kreimer et al. 2015), addressed this limitation by assessing HPV outcomes at the 12m visit or later, the first visit at which women in the different dose groups may have had an equal chance of attending. The limitation of this later analysis was that loss to follow-up at 12m was higher in the 1d group than in the 2d or 3d groups. This could have again introduced bias; however, the VE was calculated within each dose group compared to the HAV group, controlling for the differential likelihood of HPV detection due to visit attendance. The dose groups and their control groups had very similar prevalence of the different reasons for non-completion and study visit attendance and were balanced with respect to other confounders measured, leading us to believe the VEs of each dose group are unbiased. When we compare the VEs of the different dose groups we may be comparing slightly different populations. The 1d VE was calculated in a group of trial enrolees who did not attend every visit and may, on average, have lower health-seeking behaviour and be less healthy than the population who attended all study visits). Conversely, the 3d VE was calculated in a group of trial participants who attended all study visits and could be healthier on average than the 1d group (the "healthy vaccinee" effect). If these imbalances between the trial groups were borne in reality, we would expect a lower VE in the 1d arm; however, even in the presence of this potential bias, the VE of 1d is still high. |
| Misclassification | Misclassification of the exposure (the number of vaccine doses received) is unlikely across all analyses as the vaccine was not freely available to trial participants outside of the studies. However, none of the articles mention whether there was any verification of vaccination status at FU visits. All studies used highly sensitive HPV assays and standardized assays for the assessment of IgG. Misclassification of HPV incident or persistent infection is possible if HPV is simply undetectable within the cervix at the time of sampling yet latently infecting the epithelial cells. Alternatively, when looking at single timepoint detection, misclassification could result from transient HPV deposition (for example, after unprotected sexual contact) that does not result in infection. These are unavoidable problems given the limitations of HPV sampling techniques and would likely be non-differential across comparison groups. |
| Statistical analysis | Appropriate comparisons were made among CVT and PATRICIA trial participants using the HAV control group. It is legitimate to restrict analysis to those who are HPV negative at enrolment given that the primary target group for HPV vaccination is young girls prior to sexual debut (and thus prior to HPV exposure). |
| Generalizability | The trial recruited generally healthy, HIV-negative young women with few exclusion criteria and were therefore relatively pragmatic and representative of the general population. However, trial participants are, in general, healthier and less heterogenous than the general population. |
| **IARC India Vaccine Study and Sub-Study (15-19)** | |
| Selection bias | In the IARC India vaccine trial, the number of vaccine doses a participant received was dependent on her time of enrolment onto the study. It is unlikely that time of enrolment would have significantly affected the distribution of relevant confounders between the groups (e.g., their risk of HPV exposure). The 3d group was, on average, slightly poorer, potentially predisposing them to poorer HPV infection outcomes and poorer immunogenicity. However, the 3d and 1d groups had similar rates of non-vaccine type infection (excluding types 31, 33, 45) over the full period of follow-up. The IARC 10-year analyses were adjusted for predictors of non-vaccine type HPV infection. It is not clear how participants were selected for inclusion in the sub-study evaluating oral HPV infection, and thus it is difficult to assess for risk of selection bias. There were some differences in baseline characteristic between groups included in the sub-study, for example in education level and number of pregnancies, which may have been associated with HPV infection risk. There was also a trend for differences in the prevalence of non-vaccine type oral HPV infections across groups. |
| Retention/survival bias | The lack of a control group in the early analyses of the India vaccine trial makes differential rates of loss to follow-up across comparison groups a problem. At M36, 75% of the 1d group remained in follow-up, compared to 88% of the 3d group. No analysis of whether those loss to follow-up were different with respect to baseline characteristics is available in the published articles. Differential loss to follow-up could decrease the rate of HPV detection in the 1d arm simply because the cervical sample was not available, which therefore biases the VE estimate higher than the true value. However, in the later analysis with follow-up to 48m, retention rates had become more similar (75% in the 1d group vs. 78% in the 3d group), reducing the risk of survival bias when comparing VE across groups. |
| Misclassification | Misclassification of the exposure is unlikely across all analyses as the vaccine was not freely available to trial participants outside of the studies. However, none of the articles mention whether there was any verification of vaccination status at follow-up visits. All studies used highly sensitive HPV assays and standardized assays for the assessment of IgG. As above, misclassification is theoretically possibly if an early HPV infection was undetectable at the time of sampling, but this was unlikely to be differential across groups. |
| Statistical analysis | The later analysis of the India vaccine trial was improved with the enrolment of an unvaccinated control group, allowing comparison of HPV infection outcomes and controlling for visit attendance. Marriage and sexual activity may have influenced both the sampling time points for HPV infection (6m after first delivery or 18m after marriage) and risk of HPV acquisition (due to exposure), so the control group of unvaccinated married women is necessary to control for confounding by sexual activity. In the 10-year analyses, the authors adjusted VE estimates by a disease risk score that was calculated based on predictors of non-vaccine type HPV infection (excluding HPV 31, 33 and 45) (17). The number of infection events detected in the oral sub-study were very small and thus the comparison across groups was under-powered. Presence of oral HPV infection at the time of vaccination could not be ruled out. |
| Generalizability | The trial recruited generally healthy, HIV-negative young women with few exclusion criteria and were therefore relatively pragmatic and representative of the general population. However, trial participants are, in general, healthier and less heterogenous than the general population. |
| **KEN-SHE trial (1)** | |
| Selection bias | The KEN-SHE trial randomised participants to receive either 1d of the 2vHPV vaccine, 1d of the 9vHPV vaccine or 1d of a control (meningococcal) vaccine, minimising risk of bias or confounding due to differences between groups. At baseline, the groups were similar in terms of several characteristics that might be associated with risk of HPV acquisition, including age, education level and sexual behaviour. Furthermore, the baseline prevalence of various STIs was similar across groups, as was the cumulative incidence of chlamydia, gonorrhoea and non-vaccine-type HPV infections over follow-up. |
| Retention/survival bias | The double-blind randomised trial design may have reduced the risk of follow-up bias. Retention was very high, and neither retention nor completeness of endpoint swab collection differed across groups. |
| Misclassification | Misclassification of the exposure is unlikely. The authors reported that all participants received their assigned vaccine without administration error. Whilst HPV vaccine is available in Kenya, the trial participants were older than the target age for vaccination in the country so were unlikely to have received additional doses outside of the study. The trial used standardized assays for HPV detection, reducing risk of misclassification of the outcome. As above, misclassification is theoretically possibly if an early HPV infection was undetectable at the time of sampling, but this was unlikely to be differential across groups. Of note, 6% of samples collected for HPV detection were self-collected vaginal swabs rather than the ‘gold standard’ cervical swabs, potentially introducing measurement error. However, vaginal swabs have previously been reported perform similarly to cervical swabs for HPV detection and, in a post hoc analysis, the authors measured very similar VE when restricting results to those from cervical samples. |
| Statistical analysis | Analyses were performed among the ITT cohort, which is appropriate. The investigators computed VE by comparing participants who received 1d of the 2vHPV or 9vHPV with those who received 1d of the control vaccine. The main analyses were restricted to participants who were negative for type-specific HPV DNA at baseline and at M3, and negative for type-specific antibody negative at baseline. The trial was adequately powered to measure vaccine efficacy. |
| Generalizability | The trial enrolled girls and women aged 15-20 years who were sexually active, with 1-5 lifetime sexual partners (allowing measurement of efficacy within a short follow-up duration). Participants were above the primary target age group for vaccination, but analyses were restricted to those who were HPV DNA and antibody negative at baseline. Based on earlier evidence, VE is likely to be at least as high in younger girls as in the age group evaluated. Trial participants are generally considered to be healthier and less heterogenous than the general population. However, baseline HPV prevalence among trial participants was similar to that observed previously among young women in Kenya. |
| **DoRIS trial (3)** | |
| Selection bias | The DoRIS trial randomised participants to receive 1d, 2d or 3d of the 2vHPV vaccine or the 9vHPV vaccine, minimising risk of bias or confounding due to differences between groups. Baseline characteristics were largely similar across the 6 study groups. The proportion of participants reporting being sexually active at baseline was slightly higher in the 3d groups, but actual numbers were very small so differences across groups were unlikely to be significant. Similarly, there were small differences across groups in baseline HPV prevalence (HR HPV and any HPV) and HPV16 and 18 seropositivity but actual numbers were small, and differences were unlikely to be significant. |
| Retention/survival bias | Retention was very high in the trial (99% at M24), and numbers of participants attending their follow-up visits were similar across groups. Thus, the risk of retention bias was very low. |
| Misclassification | Misclassification of the exposure is unlikely. One participant received the wrong vaccine type and 2 received a vaccine dose out of window; these participants were excluded from analyses. HPV vaccine was introduced in Tanzania after commencement of the trial. However, the investigators took measures to ensure that trial participants did not receive additional vaccine doses through the national programme and that any participant who did receive additional doses was identified and excluded from analyses. The study was not blinded; however, this is unlikely to have introduced measurement error in the study endpoints as these were all immunological. The trial used standardized assays for immunogenicity assessments. |
| Statistical analysis | Analyses assessed for non-inferiority of HPV16 and 18 antibody seropositivity with one vaccine dose compared to two or three doses. The statistical methods have been used and reported in previous studies and are considered appropriate and acceptable; and the trial was adequately powered for this comparison. In non-inferiority trials, ITT analyses can lead to smaller observed effects than if all participants had adhered to the protocol, increasing the risk of falsely claiming non-inferiority. The primary immunogenicity analyses were therefore done in the PP population. As in other studies, analyses were restricted to participants who were type-specific HPV DNA and antibody negative at baseline, which is considered appropriate. |
| Generalizability | The trial recruited healthy, HIV-negative girls in the primary target age range for vaccination. Few other exclusion criteria were applied, increasing representativeness. However, as above, trial participants are generally considered to be healthier and less heterogenous than the general population. |
| **Pilot study (5)** | |
| Selection bias | Participants (all HPV16 seropositive at baseline) were randomised 1:1 to receive either 1d or 0d of HPV vaccine. However, participant numbers per group were verry small (n=5), and baseline participant characteristics were not presented or compared across groups. |
| Retention/survival bias | All participants (in both the 1d and 0d groups) were followed up at all timepoints to the M6 visit and contributed samples at each visit. |
| Misclassification | The study was not blinded; however, this is unlikely to have introduced measurement error as study endpoints were all immunological. |
| Statistical analysis | The study was not designed or powered to perform statistical analyses comparing antibody responses in participants who received 1d versus 0d of HPV vaccine. |
| Generalizability | The study only included 10 participants in total, limiting the generalisability of results. Participants were aged 27-45 years (above the primary target age for HPV vaccination) and HPV16 seropositive at baseline (per the focus of the pilot study). |

Abbreviations: CVT, Costa Rica vaccine trial; d, dose; DNA, deoxyribonucleic acid; HAV, hepatitis A vaccine; HIV, human immunodeficiency virus; HPV, human papillomavirus; IARC, International Agency for Research on Cancer; IgG, immunoglobulin G; LTFU, long-term follow-up; m, month(s); PATRICIA, PApilloma TRIal against Cancer In young Adults Trial; VE, vaccine efficacy.

**Supplementary Table 5:** Sampling, laboratory methods and analyses performed and reported by each study/article for infection outcome measures.

| **Reference / study** | **Sampling** | **Methods** | **Outcome measures reported (measure / unit)^a^** | **Outcome definition** | **Analytical cohort** | **HPV types evaluated** |
| --- | --- | --- | --- | --- | --- | --- |
| Kreimer 2011  / CVT (6) | Cervical cell samples collected from sexually experienced women at enrolment, M6, and then annually (from M0) for 4y. | SPF10 PCR DEIA^b^ and LiPA25^c^ | Incident 6m persistent infection (% risk, 95%CI) | New infection detected at M6 or later and persisting for ≥4m, confirmed by 2 samples collected ≥4m apart and testing positive for the same HPV type, with no intervening negative tests | Women who were HPV16 and HPV18 DNA negative at enrolment, and who attended follow-up visits post-enrolment | HPV16/18  HPV31/33/45 |
|  |  |  | Incident 12m persistent infection (% risk, 95%CI) | New infection detected at M6 or later and persisting for ≥10m (as above, with samples collected ≥10m apart) |  |  |
| Kreimer 2015  / CVT & PATRICIA (12) | CVT cohort: As described above for Kreimer 2011.  PATRICIA cohort: Cervical samples collected from sexually experienced women at enrolment and biannually thereafter for 4y. | As above | One-time first incident infection (% rate, 95%CI) | All first detectable infections occurring from M12, accumulated up to Y4 | M-TVC: Women who were type-specific DNA-negative at enrolment  TVC-naïve: Women who, at enrolment, were DNA-negative for 14 HR HPV types, HPV16/18 seronegative and cytology negative | HPV16/18  HPV31/33/45 |
|  |  |  | Incident 6m persistent infection (% rate, 95%CI) | New infection detected at M12 or later and persisting for ≥6m, confirmed by 2 samples collected ≥150d apart testing positive for the same HPV type, with no intervening negative tests |  |  |
|  |  |  | Incident 12m persistent infection (% rate, 95%CI) | New infection detected at M12 or later and persisting for ≥12m (as above, with samples collected >300d apart) |  |  |
| Safaeian 2018  / CVT LTFU (8) | Vaccinated cohort: As described above for Kreimer 2011. Thereafter, samples collected biennially up to Y7 from all women in follow-up study.  Unvaccinated cohort: Cervical cell samples collected biennially from Y4. | As above | One-time incident infection (% risk, 95%CI) | New infection detected at Y7 that was not present at Y4 | Women who were type-specific HPV DNA negative at Y4 | HPV16/18  HPV31/33/45  Other non-vaccine-type, oncogenic  Other non-vaccine-type, non-oncogenic |
|  |  |  | Cumulative incident infection (% risk, 95%CI) | New infection detected between M12 and Y7 | Women who were type-specific HPV DNA negative at enrolment |  |
|  |  |  | One-time prevalent infection (%, 95%CI) | Any infection detected at Y7 | All women tested (including unvaccinated cohort) |  |
| Kreimer 2020  / CVT LTFU (9) | As described above for Safaeian 2018, with follow-up to Y11. | SPF10 PCR DEIAb and LiPA25^c^ and  TypeSeq PCR^d^ | Prevalent infection (%, 95%CI) | Any infection detected at Y9 and/or Y11 | Women who attended and provided samples at the Y9 and Y11 visits | HPV16/18  Other non-vaccine-type, oncogenic  Other non-vaccine-type, non-oncogenic |
|  |  |  | One-time incident infection (% risk, 95%CI) | New infection detected at Y11 that was not present at Y9 | Women who attended and provided samples at the Y9 and Y11 visits |  |
| Tsang 2020  / CVT LTFU (10) | As described above for Safaeian 2018, with follow-up to Y11. | As above | Prevalent infection (%, 95%CI) | Any infection detected at a given study visit (Y1, Y2, Y3, Y4, Y7, Y9 or Y11) | - | HPV16/18  HPV31/33/45  Other non-vaccine-type, oncogenic  Other non-vaccine-type, non-oncogenic |
|  |  |  | One-time incident infection (% rate & % risk, 95%CI) | New infection detected at a given study visit that was not present at the prior visit | Women who were type-specific HPV DNA negative at the preceding visit |  |
|  |  |  | Incident 6m persistent infection (% rate & % risk, 95%CI) | New infection that is also detected at any visit >150d later, with no intervening negative tests | Women who were type-specific HPV DNA negative at the preceding visit |  |
| Tsang 2022  / CVT LTFU (11) | As described above for Kreimer 2011. | SPF10 PCR DEIA^b^ and LiPA25^c^ | Incident 6m persistent infection (% risk, 95%CI) | New infection detected from Y2-Y4 and persisting for ≥6m, confirmed by 2 samples collected ≥6m apart and testing positive for the same HPV type, with no intervening negative tests | Women who received 1 or 3 HPV vaccine doses and who were HPV16 DNA negative from M0 to Y2, stratified by M0 HPV16 serostatus | HPV16 |
| Sankaranarayanan 2016  / IARC India study (15) | Cervical cell samples collected 18m after marriage or 6m after first childbirth^e^ and annually thereafter for 3 consecutive years. | HPV type-specific E7 PCR bead-based multiplex genotyping | Cumulative first incident infection (% risk, 95%CI) | First detectable new infection at any visit | Married women with at least 1 cervical sample tested | HPV16/18  HPV6/11  HPV31/33/45  Other non-vaccine-type, oncogenic |
|  |  |  | Incident 12m persistent infection (% risk, 95%CI) | New type-specific HPV DNA on repeated cervical samples with a 12m interval | Married women with at least 2 cervical samples collected 12m apart |  |
| Sankaranarayanan 2018  / IARC India study (16)^f^ | Vaccinated cohort: As described above for Sankaranarayanan 2016, with ongoing sample collection to Y7.  Unvaccinated cohort: Cervical samples collected at enrolment and annually thereafter for up to 4 collections. | As above | Age-standardised^g^ cumulative incident infection (% risk, 95%CI) | All detectable infections at any visit up to Y7 | Married women with at least 1 cervical sample tested | HPV16/18  HPV31/33/45  Other non-vaccine-type, oncogenic |
|  |  |  | Incident 12m persistent infection (% risk, 95%CI) | New type-specific HPV DNA on repeated cervical samples with a ≥12m interval | Married women with at least 2 cervical samples collected 12m apart |  |
| Basu 2021  / IARC India study (17)^f^ | As described above for Sankaranarayanan 2016 and 2018, with ongoing sample collection to Y10. | As above | Cumulative incident infection (% risk, 95%CI) | All detectable infections at any visit up to Y11 | Married women with at least 1 cervical sample tested | HPV16/18  HPV6/11  HPV31/33/45  Other non-vaccine-type, oncogenic |
|  |  |  | Incident 10m persistent infection (% risk, 95%CI) | New type-specific HPV DNA on repeated cervical samples with a ≥10m interval | Married women with at least 2 cervical samples collected 10m apart |  |
| Gheit 2023  / IARC India sub-study (19) | Deep oral gargle specimens collected from participants  enrolled from June 2015 to October 2016 and eligible for collection of cervical cells (as described above). Time point(s) for collection not stated. | E7-MPG  PCR and Luminex-based assay^h^ | Age-standardised^g^ prevalent infection (%, 95%CI) | Not defined | Not defined | HPV16/18  HPV6/11  HPV31/33/45  Other non-vaccine-type, oncogenic |
| Barnabas 2022  / KEN-SHE (1) | Cervical and external genital swabs collected at enrolment. Self-collected vaginal swabs obtained at M3. Cervical swabs collected at M6, M12 and M18. | Anyplex II HPV28 assay^i^ | Incident 6m persistent infection (% rate, 95%CI) | New type-specific HPV infection detected at 2 consecutive timepoints ≥4m apart, occurring after M3 | Women who were type-specific HPV DNA negative at enrolment and M3, and HPV antibody negative at enrolment | HPV16/18  Other vaccine-type, oncogenic |

Abbreviations: CI, confidence interval; CVT, Costa Rica Vaccine Trial; d, days; DEIA, direct enzyme immunoassay; DNA, deoxyribonucleic acid ; FU, follow-up; HPV, human papillomavirus; HR, high-risk; IARC, International Agency for Research on Cancer; LTFU, long-term follow-up; M/m, month/months; PCR, polymerase chain reaction; TVC: total vaccinated cohort; M-TVC: modified TVC; Y/y, year/years.

^a^Incidence risk denotes the number of new cases occurring per population at risk (i.e., using the number of women in the analytical population as the denominator). Incidence rate denotes the number of new cases per population at risk in a given time period (i.e., using person-years as the denominator).

^b^SPF10 PCR DEIA: SPF10 PCR primer system and DNA enzyme immunoassay detection of amplimers (DDL Diagnostic Laboratory, Voorburg, the Netherlands).

^c^LiPA25: HPV line probe assay containing probes for 25 HPV genotypes (Labo Biomedical Products, Rijswijk, the Netherlands).

^d^US National Cancer Institute's in-house assay that detects 51 HPV genotypes.

^e^Whichever occurred earlier.

^f^In addition to the HPV infection evaluations described in the table, married women in the vaccinated and unvaccinated groups (with the latter including the second control group) were invited to undergo cervical screening when they reached 25 years of age. Women were first tested for HPV infection using Hybrid Capture II (HC-II; Qiagen, Gaithersburg, MD, USA). Where HC-II was positive, women were recalled for colposcopy, and Digene HPV genotyping PS test (Qiagen) was used to determine the genotype associated with any detected cervical neoplasia.

^g^Age-standardisation used the direct standardisation method and the 2011 Indian female population as the standard population.

^h^A multiplex PCR and Luminex based assay (Luminex Corp., Austin, TX).

^i^A multiplex real-time PCR (Seegene, Seoul, South, Korea).

**Supplementary Table 6:** Vaccine-type infection results from articles that compared one dose of the 4vHPV or 9vHPV vaccine to either no HPV vaccination or multidose schedules.

| **Reference / study** | **Follow-up duration** | **Infection outcome^a^** | **3-dose HPV arm** | | **2-dose HPV arm^b^** | | **1-dose HPV arm** | | **Control arm^c^** | | **RR or PR (95%CI), *p value*^d^** | | |
| --- | --- | --- | --- | --- | --- | --- | --- | --- | --- | --- | --- | --- | --- |
|  |  |  | **Events/N** | **%**  **(95%CI)^d^** | **Events/N** | **%**  **(95%CI)^d^** | **Events/N** | **%**  **(95%CI)^d^** | **Events/N** | **%**  **(95%CI)^d^** | **1 dose/**  **3 doses^e^** | **1 dose/**  **2 doses^e^** | **1 dose/ control** |
| **Merck 4vHPV** | | | | | | | | | | | | | |
| **Incident HPV6/11/16/18 infection** | | | | | | | | | | | | | |
| Sankaranarayanan 2016  / IARC India study (15) | Median: 4.7y  IQR: 4.2-5.1y | Cumulative 1st incident | 3/536 | 0.6  (0.1-1.6) | 5/526 | 1.0  (0.3-2.2) | 14/870 | 1.6  (0.9-2.7) | - | - | 2.9 (0.8-10.0)  *0.13* | 1.7 (0.6-4.7)  *0.35* | - |
| Basu 2021  / IARC India study (17) | Median:9.0y  IQR: 8.2-9.6y | One-time incident | 110/2,019 | 5.4  (4.5-6.5) | 107/2,166 | 4.9  (4.1-5.9) | 154/2,858 | 5.4  (4.6-6.3) | 192/1,479 | 13.0  (11.3-14.8) | 0.9 (0.8-1.3)  *0.95* | 1.1 (0.9-1.4)  *0.52* | 0.4 (0.3-0.5)  *<0.01* |
| **Persistent HPV6/11/16/18 infection^f^** | | | | | | | | | | | | | |
| Basu 2021  / IARC India study (17) | Median:9.0y  IQR: 8.2-9.6y | 10m persistent | 2/1,460 | 0.1  (0.0-0.5) | 1/1,452 | 0.1  (0.0-0.4) | 2/2,135 | 0.1  (0.0-0.3) | 35/1,260 | 2.8  (1.9-3.8) | 0.7 (0.1-4.8)  *1.00* | 1.4 (0.1-15.0)  *1.00* | 0.0 (0.0-0.1)  *<0.01* |
| **Prevalent oral HPV6/11/16/18 infection** | | | | | | | | | | | | | |
| Gheit 2023  / IARC India sub-study (19)^g^ | Not reported | Not reported | 15/323 | 4.6  (2.6-7.5) | 6/190 | 3.2  (1.2-6.7) | 14/204 | 6.9  (3.8-11.2) | 15/179 | 8.4  (4.8-13.4) | 1.5 (0.7-3.0)  *0.33* | 2.2 (0.9-5.5)  *0.11* | 0.8 (0.4-1.6)  *0.70* |
| **Merck 9vHPV** | | | | | | | | | | | | | |
| **Persistent HPV16/18/31/33/45/52/58 infection^h^** | | | | | | | | | | | | | |
| Barnabas 2022  / KEN-SHE (1) | 18m^i^ | 6m persistent | - | - | - | - | 4/325 | 1.2  (0.3-3.1) | 29/290 | 10.0  (6.8-14.0) | - | - | 0.1 (0.0-0.3)  *<0.01* |

Abbreviations: 4vHPV, quadrivalent HPV [vaccine]; 9vHPV, nonavalent HPV [vaccine]; CI, confidence interval; HPV, human papillomavirus; IARC, International Agency for Research on Cancer; IQR, interquartile range; m, month(s); N, number of participants in group; PR, prevalence ratio; RR, risk ratio; y, year.

^a^Definitions of infection outcomes used in each study are provided in Supplementary Table 5. All outcomes refer to cervical infections unless stated otherwise.

^b^Results are shown only for two-dose arms where participants received dose one at day 0 and dose two at day 180.

^c^Results are shown for unvaccinated control arms for Basu 2021 and Gheit 2023 (17, 19), and a meningococcal vaccine control arm for Barnabas 2022 (1). No control arm was used in Sankaranarayanan 2016 (15).

^d^Proportions (%), unadjusted RRs and PRs, 95%CIs and two-sided Fisher’s exact p values were calculated by the authors of this review using data provided in the included articles.

^e^In studies where participants were not specifically randomised to receive one HPV vaccine dose versus either no HPV vaccination or multidose HPV vaccination, RRs and PRs calculated for one versus two or three doses must be interpreted with caution because of potential for selection bias due to differences in follow-up between the groups.

^f^Sankaranarayanan 2016 (15) aimed to measure persistent infection but did not detect any persistent infections in any arm.

^g^Gheit 2023 (19) also reported data on the prevalence of cervical vaccine-type infections. However, these were not reported by the number of doses received, and thus results of cervical infections from this sub-study are not presented here.

^h^Barnabas 2022 (1) reported only oncogenic infection types; not HPV6 and HPV11 infections.

^i^Mean, median, IQR, or SD were not reported for this study.

**Supplementary Table 7:** HPV31/33/45 infection results from articles comparing one dose of the 2vHPV vaccine or the 4vHPV vaccine to either no HPV vaccination or multidose schedules.

| **Reference / study** | **Follow-up duration** | **Infection outcome^a^** | **3-dose HPV arm** | | **2-dose HPV arm^b^** | | **1-dose HPV arm** | | **Control arm^c^** | | **RR or PR (95%CI), *p value*^d^** | | |
| --- | --- | --- | --- | --- | --- | --- | --- | --- | --- | --- | --- | --- | --- |
|  |  |  | **Events/N** | **%**  **(95%CI)^d^** | **Events/N** | **%**  **(95%CI)^d^** | **Events/N** | **%**  **(95%CI)^d^** | **Events/N** | **%**  **(95%CI)^d^** | **1 dose/**  **3 doses^e^** | **1 dose/**  **2 doses^e^** | **1 dose/**  **control** |
| **GlaxoSmithKline 2vHPV^f^** | | | | | | | | | | | | | |
| **Incident HPV31/33/45 infection** | | | | | | | | | | | | | |
| Kreimer 2015  / CVT (12) | Mean: 4.0y  SD: 0.7y | One-time incident | 710/11,156 | 6.3  (5.8-6.7) | 55/615 | 8.9  (6.8-11.5) | 26/293 | 8.9  (5.9-12.7) | 35/253 | 13.8  (9.8-18.7) | 1.4 (1.0-2.1)  *0.09* | 1.0 (0.6-1.5)  *1.00* | 0.6 (0.4-1.0)  *0.08* |
| Safaeian 2018  / CVT LTFU (8) | Median: 6.9y  IQR: 6.5-7.3y | One-time incident | 31/2042 | 1.5  (1.0-2.1) | 0/78 | 0.0  (0.0-4.6) | 2/134 | 1.5  (0.2-5.3) | - | - | 1.0 (0.2-4.1)  *1.00* | .  *0.53* | - |
|  |  | Cumulative incident | 164/2,043 | 8.0  (6.9-9.3) | 7/79 | 8.9  (3.6-17.4) | 11/134 | 8.2  (4.2-14.2) | - | - | 1.0 (0.6-1.8)  *0.87* | 0.9 (0.4-2.3)  *1.00* | - |
| Tsang 2020  / CVT (10) | 11y^g^ | One-time incident | 24/1,339 | 1.8  (1.2-2.7) | 1/61 | 1.6  (0.0-8.8) | 1/111 | 0.9  (0.0-4.9) | 79/1,668 | 4.7  (3.8-5.9) | 0.5 (0.1-3.7)  *0.72* | 0.5 (0.0-8.6)  *1.00* | 0.2 (0.0-1.4)  *0.06* |
| **Prevalent HPV31/33/45 infection** | | | | | | | | | | | | | |
| Safaeian 2018  / CVT LTFU (8) | Median: 6.9y  IQR: 6.5-7.3y | One-time prevalent | 48/2,043 | 2.3  (1.7-3.1) | 0/79 | 0.0  (0.0-4.6) | 2/134 | 1.5  (0.2-5.3) | 132/2,382 | 5.5  (4.7-6.5) | 0.6 (0.2-2.6)  *0.77* | .  *0.53* | 0.3 (0.1-1.1)  *0.05* |
| Tsang 2020  / CVT LTFU (10) | 11y^g^ | One-time prevalent | 50/1,943 | 2.6  (1.9-3.4) | 1/74 | 1.4  (0.0-7.3) | 4/172 | 2.3  (0.6-5.8) | 139/2,204 | 6.3  (5.3-7.4) | 0.9 (0.3-2.5)  *1.00* | 1.7 (0.2-15.1)  *1.00* | 0.4 (0.1-1.0)  *0.03* |
| **Persistent HPV31/33/45 infection** | | | | | | | | | | | | | |
| Kreimer 2015  / CVT & PATRICIA (12) | Mean: 4.0y  SD: 0.7y | 6m persistent | 266/11,150 | 2.3  (2.1-2.7) | 18/615 | 2.9  (1.7-4.6) | 9/293 | 3.1  (1.4-5.8) | 15/253 | 5.9  (3.3-9.6) | 1.3 (0.7-2.5)  *0.44* | 1.0 (0.5-2.3)  *1.00* | 0.5 (0.2-1.2)  *0.14* |
|  |  | 12m persistent | 175/11,150 | 1.6  (1.3-1.8) | 11/615 | 1.8  (0.9-3.2) | 5/293 | 1.7  (0.6-3.9) | 8/253 | 3.2  (1.4-6.1) | 1.1 (0.5-2.6)  *0.81* | 1.0 (0.3-2.7)  *1.00* | 0.5 (0.2-1.6)  *0.40* |
| Tsang 2020  / CVT LTFU (10) | 11y^g^ | 6m persistent from Y9^h^ | 11/1,627 | 0.7  (0.3-1.2) | 0/62 | 0.0  (0.0-5.8) | 0/109 | 0.0  (0.0-3.3) | 29/1,839 | 1.6  (1.1-2.3) | 0.0 (CI NC)  *1.00* | .  *UTC^i^* | 0.0 (CI NC)  *0.40* |
| **Merck 4vHPV** | | | | | | | | | | | | | |
| **Incident HPV31/33/45 infection** | | | | | | | | | | | | | |
| Sankaranarayanan 2016  / IARC India study (15) | Median: 4.7y  IQR: 4.2-5.1y | Cumulative 1st incident | 32/536 | 6.0  (4.1-8.3) | 26/526 | 4.9  (3.3-7.2) | 77/870 | 8.9  (7.0-10.9) | - | - | 1.5 (1.0-2.2)  *0.05* | 1.8 (1.2-2.8)  *0.01* | - |
| Sankaranarayanan 2018  / IARC India study (16) | Up to 7y^j^ | Cumulative incident | 60/1,180 | 5.1  (3.9-6.5) | 53/1,179 | 4.5  (3.4-5.8) | 103/1,823 | 5.7  (4.6-6.8) | 114/1,481 | 7.7  (6.4-9.2) | 1.1 (0.8-1.5)  *0.56* | 1.3 (0.9-1.7)  *0.18* | 0.7 (0.6-0.9)  *0.02* |
| Basu 2021  / IARC India study (17) | Median:9.0y  IQR: 8.2-9.6y | One-time incident | 86/2,019 | 4.3  (3.4-5.2) | 89/2,166 | 4.1  (3.3-5.0) | 136/2,858 | 4.8  (4.0-5.6) | 148/1,479 | 10.0  (8.5-11.7) | 1.1 (0.9-1.5)  *0.44* | 1.2 (0.9-1.5)  *0.30* | 0.5 (0.4-0.6)  *<0.01* |
| **Persistent HPV31/33/45 infection^k^** | | | | | | | | | | | | | |
| Sankaranarayanan 2018  / IARC India study (16) | Up to 7y^j^ | 12m persistent | 1/604 | 0.2  (0.0-0.9) | 1/608 | 0.2  (0.0-0.9) | 7/959 | 0.7  (0.3-1.5) | 6/1,141 | 0.5  (0.2-1.1) | 4.4 (0.5-35.7)  *0.16* | 4.4 (0.5-36.0)  *0.16* | 1.4 (0.5-4.1)  *0.59* |
| Basu 2021  / IARC India study (17) | Median:9.0y  IQR: 8.2-9.6y | 10m persistent | 7/1,460 | 0.5  (0.2-1.0) | 11/1,452 | 0.8  (0.4-1.4) | 14/2,135 | 0.7  (0.4-1.1) | 14/1,260 | 1.1  (0.6-1.9) | 1.4 (0.6-3.4)  *0.66* | 0.9 (0.4-1.9)  *0.83* | 0.6 (0.3-1.2)  *0.17* |
| **Prevalent oral HPV31/33/45 infection** | | | | | | | | | | | | | |
| Gheit 2023  / IARC India sub-study (19) | Not reported | Not reported | 0/323 | 0.0  (0.0-1.1) | 0/190 | 0.0  (0.0-1.9) | 1/204 | 0.4  (0.0-2.7) | 0/179 | 0.0  (0.0-2.0) | .  *0.39* | .  *1.00* | .  *1.00* |

Abbreviations: 2vHPV, bivalent HPV [vaccine]; 4vHPV, quadrivalent HPV [vaccine]; CI, confidence interval; CVT, Costa Rica Vaccine Trial; HPV, human papillomavirus; IARC, International Agency for Research on Cancer; IQR, interquartile range; LTFU, long-term follow-up; m, month(s); N, number of participants in group; NC, not calculated; PR, prevalence ratio; RR, risk ratio; SD, standard deviation; UTC, unable to compute; y, years.

^a^Definitions of infection outcomes used in each study are provided in Supplementary Table 5. All endpoints refer to cervical infections unless stated otherwise.

^b^Results are shown only for two-dose arms where participants received dose one at day 0 and dose two at day 180.

^c^Results are shown for one-dose control vaccine arms for Kreimer 2015 (12) (HAV vaccine) and Barnabas 2023 (1) (meningococcal vaccine), and unvaccinated control arms for Safaeian 2018 (8) (persistent infection only), Tsang 2020 (10), Sankaranarayanan 2018 (16), Basu 2021 (17) and Gheit 2023 (19). For Kreimer 2015 (12), comparison of the one-dose HPV vaccine arm with the one-dose HAV (rather than multidose HAV) arm minimizes the potential for selection bias due to differences in follow-up. No control arm was used in Sankaranarayanan 2016 (15).

^d^Proportions (%), unadjusted RRs and PRs, 95%CIs and two-sided Fisher’s exact p values were calculated by the authors of this review using data provided in the included articles.

^e^In studies where participants were not specifically randomised to receive one HPV vaccine dose versus either no HPV vaccination or multidose HPV vaccination, RRs and PRs calculated for one versus two or three doses must be interpreted with caution because of potential for selection bias due to differences in follow-up between the groups.

^f^Kreimer 2011 (6) presented some HPV31/33/45 infection results; however, insufficient data are provided to evaluate effectiveness of one dose of HPV vaccine against these types.

^g^Median follow-up time is presented separately in this paper for Y0-4 of CVT and the subsequent long-term follow-up (to Y11). Median follow-up time was 4.5 years for all arms in CVT, and ranged from 6.3 years in the unvaccinated control arm to 6.7 years in the 3-dose arm in the long-term follow-up.

^h^Results are shown for new infections detected at Y9 that persisted for at least 150 days.

^i^STATA does not compute a p value using Fisher’s exact test where both numerators are 0.

^j^Mean, median, IQR, or SD were not reported for this study.

^k^Sankaranarayanan 2016 (15) aimed to measure persistent infection but did not detect any persistent infections in any arm.

**Supplementary Table 8:** Sampling, laboratory methods and analyses performed and reported by each study/article for immunogenicity outcome measures.

| **Reference / study** | **Sampling** | **Methods** | **Outcome measures reported (measure / unit) with definitions where applicable** | **Analytical cohort** | **HPV types evaluated** |
| --- | --- | --- | --- | --- | --- |
| Safaeian 2013  / CVT (7)^a^ | Vaccinated cohort: Serum collected at enrolment, M1, M6, Y1, Y2, Y3 and Y4.  Naturally infected cohort: Serum collected at enrolment, pre-vaccination. | HPV L1 VLP ELISA | 1. Antibody levels (GM EU/ml, 10th, 25th, 75th and 90th percentiles, 95%CI)  2. Seropositivity (% of analytical population seroconverting), using lab-defined seropositivity cut-offs of 8 EU/ml for HPV16 and 7 EU/ml for HPV18 | Women with sera available at all study visits | HPV16/18 |
| Safaeian 2018  / CVT LTFU (8) | Vaccinated cohort: Serum collected at Y4 and Y7. | HPV L1 VLP ELISA | 1. Antibody levels (GM EU/ml, 10th, 25th, 75th and 90th percentiles, 95%CI)  2. Seropositivity (% of analytical population seroconverting), using lab-defined seropositivity cut-offs of 8 EU/ml for HPV16 and 7 EU/ml for HPV18 | Women included in study above (Safaeian 2013) with sufficient sera available | HPV16/18 |
|  |  | GuHCl-modified HPV L1 VLP avidity ELISA | Antibody avidity index (GM avidity index, 95%CI, IQR) |  | HPV16 |
| Kreimer 2020  / CVT LTFU (9)^a^ | Vaccinated cohort: Serum collected at Y9 and Y11. | HPV L1 VLP ELISA | 1. Antibody levels (GM EU/ml and IU, 95%CI)  2. Seropositivity (% of analytical population seroconverting), using lab-defined seropositivity cut-offs of 8 EU/ml for HPV16 and 7 EU/ml for HPV18 | Women who attended and provided  samples at Y9 and Y11 | HPV16/18 |
| Tsang 2022  / CVT LTFU (11) | Vaccinated cohort: Serum collected at enrolment, Y1, Y2, Y3, Y4, Y7, Y9 and Y11. | GuHCl-modified HPV L1 VLP avidity ELISA | Antibody avidity index (GM avidity index, 95%CI, IQR) | Women with HPV16 IgG ELISA results available^b^ and with ≥2 serum samples collected from Y1 to Y11, stratified by M0 HPV16 serostatus | HPV16 |
| Sankaranarayanan 2016  / IARC India study (15)^a^ | Plasma collected at enrolment and M7, Y1, Y1.5, Y2, Y3, Y4 and Y5 | Luminex-based multiplex binding assay^c^ | 1. Antibody levels (GM MFI, 95%CI)  2. Seropositivity (% of analytical population seroconverting), using seropositivity cut-offs of 100 MFI for HPV16, 41 MFI for HPV18, 240 MFI for HPV6 and 48 MFI for HPV11 | Convenience sample | HPV16/18  HPV6/11 |
|  |  | Modified HPV L1 genotype-specific binding antibody assay (M7 and Y1.5 only) | Antibody avidity index (GM avidity index, 95%CI) |  |  |
| Sankaranarayanan 2018  / IARC India study (16)^a^ | Plasma collected at enrolment and M7, Y1, Y1.5, Y2, Y3, Y4 and Y5 | Luminex-based multiplex binding assay^c^ | 1. Antibody levels (GM MFI, 95%CI)  2. Seropositivity (% of analytical population seroconverting), using seropositivity cut-offs of 100 MFI for HPV16 and 41 MFI for HPV18 | Convenience sample | HPV16/18 |
|  |  | Modified HPV L1 genotype-specific binding antibody assay (M7 and Y1.5 only) | Antibody avidity index (GM avidity index, 95%CI) |  |  |
| Joshi 2023  / IARC India study (18)^a^ | Vaccinated cohort: Plasma collected at enrolment and M7, Y1, Y1.5, Y2, Y3, Y4, Y5 and Y10  Unvaccinated cohort: Plasma collected at single time point | Luminex-based multiplex binding assay^c^ | 1. Antibody levels (GM IU for HPV16/18 and AU/ml for HPV6/11, 95%CI)  2. Seropositivity (% of analytical population seroconverting), using seropositivity cut-offs of 1.0 IU/ml for HPV16, 0.3 IU/ml for HPV18, 0.2 AU/ml for HPV6 and 0.4 AU/ml for HPV11 | Women from convenience sample with available samples at least from enrolment and Y1 | HPV16/18  HPV6/11 |
| Scherer 2016  / Pilot study (5)^a^ | Plasma collected 6m prior to vaccination, on day of vaccination, and at week 1, M1 and M6. | Anti-L1 binding assay using GST-HPV L1 fusion proteins on BioPlex with magnetic beads | 1. Antibody levels (MFI converted to U/ml)  2. Seropositivity, using seropositivity cut-off of 3 U/ml | All women included in the study | HPV16 |
| Watson-Jones 2022  / DoRIS (3) | Serum collected at enrolment, M1, M7, Y1 and Y2. | HPV L1 VLP ELISA | 1. Antibody levels (GM IU, 95%CI)  2. Seropositivity (% of analytical population seroconverting), using seropositivity cut-offs of 1·309 IU/mL for HPV16 and 1·109 IU/mL for HPV18 | Girls who were type specific HPV seronegative and DNA negative at enrolment | HPV16/18 |
|  |  | GuHCl-modified HPV L1 VLP avidity ELISA | Antibody avidity index (GM avidity index, 95%CI) |  |  |

Abbreviations: AU, arbitrary unit; CI, confidence interval; CVT, Costa Rica Vaccine Trial; ELISA, enzyme-linked immunosorbent assay; EU, ELISA unit; GM, geometric mean; GST, glutathione-S-transferase; GuHCl, guanidine hydrochloride; HPV, human papillomavirus; IARC, International Agency for Research on Cancer; IgG, immunoglobulin G; IU, international unit; IQR, interquartile range; LTFU, long-term follow-up; M, month; MFI, mean fluorescent intensity; ml, millilitre; VLP, virus-like particle; w, week; Y, year.

^a^Safaeian 2013 (7), Kreimer 2020 (9), Sankaranarayanan 2016 (15), Sankaranarayanan 2018 (16), Joshi 2023 (18) and Scherer 2016 (5) examined neutralising antibody responses, and Scherer 2016 additionally examined memory B cell responses. However, these evaluations are outside the remit of this systematic review and/or paper, and thus associated sampling, methods and analyses are not presented here.

^b^Necessary to control for the concentration of antibody added to the avidity assay.

^c^Whilst sampling was performed at all timepoints, results are only shown in the articles for selected time points. In Sankaranarayanan 2016 (15), binding antibody results are shown at enrolment, M7, M12, M18, M36 and M48 for the two-dose and three-dose arms, and at M12, M18 and M36 for the one-dose arm. Results are extended to M48 in the one-dose arm in Sankaranarayanan 2018 (16). In Joshi 2023 (18), results are shown at enrolment, M7 (three-dose arm only), M12 (one-dose arm only), M18, M36 and M120.

**Supplementary Table 9:** HPV6 and HPV11 seropositivity and geometric mean antibody level results from articles evaluating one versus two or three doses of the 4vHPV vaccine.

| **Reference / study** | **Time**  **point** | **# seropositive^a^/N participants (% Seropositive, 95%CI^b^)** | | | **GM concentrations/MFI (95%CI)** | | |
| --- | --- | --- | --- | --- | --- | --- | --- |
|  |  | **3 doses** | **2 doses^c^** | **1 dose** | **3 doses** | **2 doses^c^** | **1 dose** |
| **Merck 4vHPV^d^** | | | | | | | |
| **HPV6** | | | | | | | |
| Sankaranarayanan 2016  / IARC India study (15) | M0 | 51/1,000  5.1 (3.8-6.7) | 44/937  4.7 (3.4-6.3) | - | MFI 24  (22-26) | MFI 26  (24-29) | - |
|  | M7 | 308/308  100.0 (98.8-100.0) | 317/317  100.0 (98.8-100.0) | - | MFI 4,715  (4,484-4,957) | MFI 4,922 (4,675-5,182) | - |
|  | Y1 | - | - | 192/528  36.4 (32.3-40.6) | - | - | MFI 167  (153-183) |
|  | Y1.5 | 296/313  94.6 (91.4-96.8) | 292/314  93.0 (89.6-95.6) | 182/476  38.2 (33.8-42.8) | MFI 986  (900-1,080) | MFI 830  (756-911) | MFI 169  (154-186) |
|  | Y3 | 227/271  83.8 (78.8-87-9) | 210/278  75.5 (70.0-80.5) | 138/510  27.1 (23.2-31.1) | MFI 623  (556-699) | MFI 472  (420-530) | MFI 131  (120-143) |
| Joshi 2023  / IARC India study (18) | M7 | 154/154  100.0 (97.6-100.0) | - | - | 305 AU/ml  (264-352) | - | - |
|  | Y1 | - | - | 150/150  100.0 (97.6-100.0) | - | - | 3 U/ml  (3-3) |
|  | Y1.5 | 154/154  100.0 (97.6-100.0) | - | 148/148  100.0 (97.5-100.0) | 29 AU/ml  (24-34) | - | 2 AU/ml  (2-3) |
|  | Y3 | 136/136  100.0 (97.3-100.0) | - | 149/150  99.3 (96.3-100.0) | 17 AU/ml  (14-21) | - | 2 AU/ml  (2-3) |
|  | Y10 | 167/167  100.0 (97.8-100.0) | 190/190  100.0 (98.1-100.0) | 314/324  96.9 (94.4-98.5) | 8 AU/ml  (7-9) | 6 AU/ml  (5-7) | 2 AU/ml  (2-2) |
| **HPV11** | | | | | | | |
| Sankaranarayanan 2016  / IARC India study (15) | M0 | 56/1,000  5.6 (4.3-7.2) | 43/937  4.6 (3.3-6.1) | - | MFI 6  (6-7) | MFI 7  (6-7) | - |
|  | M7 | 308/308  100.0 (98.8-100.0) | 317/317  100.0 (98.8-100.0) | - | MFI 6,163  (5,909-6,427) | MFI 6,905  (6,622-7,200) | - |
|  | Y1 | - | - | 466/528  88.3 (85.2-90.9) | - | - | MFI 163  (149-179) |
|  | Y1.5 | 313/313  100.0 (98.8-100.0) | 314/314  100.0 (98.8-100.0) | 427/476  89.7 (86.6-92.3) | MFI 1,327  (1,216-1,449) | MFI 1,328 (1,223-1,443) | MFI 164  (148-180) |
|  | Y3 | 268/271  98.9 (96.8-99.8) | 277/278  99.6 (98.0-100.0) | 407/510  79.8 (76.1-83.2) | MFI 683  (609-765) | MFI 653  (585-729) | MFI 122  (111-135) |
| Joshi 2023  / IARC India study (18) | M7 | 154/154  100.0 (97.6-100.0) | - | - | 383 AU/ml (331-444) | - | - |
|  | Y1 | - | - | 147/150  98.0 (94.3-99.6) | - | - | 4 AU/ml  (3-5) |
|  | Y1.5 | 154/154  100.0 (97.6-100.0) | - | 140/148  94.6 (89.6-97.6) | 37 AU/ml  (31-43) | - | 3 AU/ml  (3-4) |
|  | Y3 | 136/136  100.0 (97.3-100.0) | - | 142/150  94.7 (89.8-97.7) | 21 AU/ml  (17-25) | - | 3 AU/ml  (3-4) |
|  | Y10 | 167/167  100.0 (97.8-100.0) | 188/190  98.9 (96.2-99.9) | 303/324  93.5 (90.3-95.9) | 10 AU/ml  (9-12) | 10 AU/ml  (9-12) | 3 AU/ml  (3-4) |

Abbreviations: 4vHPV, quadrivalent HPV [vaccine]; CI, confidence interval; GM, geometric mean; HPV, human papillomavirus; IARC, International Agency for Research on Cancer; IU, international unit; M, month; MFI, median fluorescence intensity; ml, millilitre; Y, year.

^a^Definitions of seropositivity used in each study are provided in Supplementary Table 8.

^b^Seropositivity proportions (%) and 95%CI were calculated by the authors of the systematic review using data provided in the included articles.

^c^Results are shown only for two-dose arms where participants received dose one at day 0 and dose two at day 180.

**Supplementary Table 10:** HPV antibody avidity results from articles evaluating one versus two or three doses of HPV vaccine.

| **Reference / study** | **Time**  **point** | **HPV16** | | | | | | | | | | **HPV18 GM avidity index (95%CI)** | | | | | | |
| --- | --- | --- | --- | --- | --- | --- | --- | --- | --- | --- | --- | --- | --- | --- | --- | --- | --- | --- |
|  |  | **3 doses** | | | **2 doses^a^** | | | **1 dose** | | | | **3 doses** | | | **2 doses^a^** | | **1 dose** | |
|  |  | **N** | **GM avidity index (95%CI)** | | **N** | **GM avidity index (95%CI)** | | **N** | **GM avidity index (95%CI)** | | | **N** | **GM avidity index (95%CI)** | | **N** | **GM avidity index (95%CI)** | **N** | **GM avidity index (95%CI)** |
| **2vHPV vaccine** | | | | | | | | | | | | | | | | | | |
| Safaeian 2018  / CVT LTFU (8) | Y4 | 165 | 2.5 (2.4-2.6) | | 61 | 2.3 (2.1-2.6) | | 104 | 2.0 (1.8-2.2) | | | - | - | | - | - | - | - |
|  | Y7 | 165 | 2.5 (2.4-2.6) | | 61 | 2.3 (2.1-2.6) | | 104 | 2.0 (1.8-2.2) | | | - | - | | - | - | - | - |
| Tsang 2022  / CVT LTFU (11)^b^ | Y1 | 161 | 2.9 (2.8-2.9) | | - | - | | 52 | 2.4 (2.1-2.7) | | | - | - | | - | - | - | - |
|  | Y2 | 142 | 3.0 (2.9-3.1) | | - | - | | 56 | 2.6 (2.4-2.8) | | | - | - | | - | - | - | - |
|  | Y3 | 119 | 3.0 (2.9-3.1) | | - | - | | 51 | 2.8 (2.7-3.0) | | | - | - | | - | - | - | - |
|  | Y4 | 191 | 3.0 (3.0-3.1) | | - | - | | 123 | 2.8 (2.6-2.9) | | | - | - | | - | - | - | - |
|  | Y7 | 225 | 3.1 (3.0-3.1) | | - | - | | 162 | 2.8 (2.7-2.9) | | | - | - | | - | - | - | - |
|  | Y9 | 136 | 3.1 (3.0-3.1) | | - | - | | 125 | 2.7 (2.6-2.9) | | | - | - | | - | - | - | - |
|  | Y11 | 141 | 3.0 (2.9-3.1) | | - | - | | 178 | 2.7 (2.6-2.8) | | | - | - | | - | - | - | - |
| Watson-Jones 2022  / DoRIS (3) | Y1 | 141 | 3.0 (2.9-3.0) | | 140 | 2.8 (2.8-2.9) | | 147 | 2.7 (2.7-2.8) | | | 136 | 1.8 (1.7-1.8) | | 139 | 1.7 (1.7-1.8) | 140 | 1.6 (1.5-1.6) |
|  | Y2 | 141 | 3.1 (3.0-3.1) | | 141 | 3.0 (2.9-3.0) | | 148 | 3.0 (2.9-3.0) | | | 136 | 1.8 (1.8-1.9) | | 140 | 1.8 (1.7-1.8) | 141 | 1.7 (1.6-1.8) |
| **4vHPV vaccine^c^** | | | | | | | | | | | | | | | | | | |
| Sankaranarayanan 2016  / IARC India study (15)^d^ | M7 | 97 | 70 (68-73) | | 99 | 65 (62-68) | | - | - | | | 97 | 82 (79-85) | | 99 | 80 (75-85) | - | - |
|  | Y1.5 | 136 | 67 (64-71) | | 139 | 66 (63-70) | | 130 | 74 (68-80) | | | 136 | 76 (73-81) | | 139 | 75 (71-80) | 130 | 85 (77-93) |
| **9vHPV vaccine** |  |  | |  | | |  | | |  |  | | |  | | | | |
| Watson-Jones 2022  / DoRIS (3) | Y1 | 140 | 2.7 (2.7-2.8) | | 142 | 2.9 (2.8-2.9) | | 145 | 2.6 (2.5-2.7) | | | 142 | 2.0 (1.9-2.0) | | 137 | 2.0 (2.0-2.1) | 136 | 1.9 (1.9-2.0) |
|  | Y2 | 140 | 2.9 (2.8-2.9) | | 141 | 2.9 (2.9-3.0) | | 145 | 2.9 (2.8-2.9) | | | 142 | 2.0 (2.0-2.0) | | 136 | 2.1 (2.0-2.1) | 136 | 2.0 (1.9-2.1) |

Abbreviations: 2vHPV, bivalent HPV [vaccine]; 4vHPV, quadrivalent HPV [vaccine]; 9vHPV, nonavalent HPV [vaccine]; CI, confidence interval; CVT, Costa Rica Vaccine Trial; GM, geometric mean; HPV, human papillomavirus; IARC, International Agency for Research on Cancer; LTFU, long-term follow-up; M, month; Y, year.

^a^Results are shown only for two-dose arms where participants received dose one at day 0 and dose two at day 180.

^b^Results are shown for participants who were HPV16 seronegative at first HPV vaccination. The paper also provides data for participants who were HPV16 seropositive at first HPV vaccination; these are not presented here.

^c^Sankaranarayanan 2018 (16) also presented brief results on antibody avidity, but these repeated results were already presented in Sankaranarayanan 2016 (15); no additional results were reported.

^d^At M18, HPV6 avidity index was 70 (95%CI 66-74) for the three-dose arm, 68 (95%CI 64-73) for the two-dose arm, and 65 (95%CI 59-72) for the one-dose arm. HPV11 avidity index was 85 (95%CI 81-89) for the three-dose arm, 84 (95%CI 80-89) for the two-dose arm, and 88 (95%CI 80-97) for the one-dose arm. Numbers of participants per arm were the same as those provided in the table for the HPV16 and HPV18 results at the Y1.5 timepoint.

**REFERENCES**

1. Barnabas RV, Brown ER, Onono MA, Bukusi EA, Njoroge B, Winer RL, et al. Efficacy of single-dose HPV vaccination among young African women. NEJM Evid. 2022;1(5):EVIDoa2100056.

2. Barnabas RV, Brown ER, Onono M, Bukusi EA, Njoroge B, Winer RL, et al. Single-dose HPV vaccination efficacy among adolescent girls and young women in Kenya (the KEN SHE Study): study protocol for a randomized controlled trial. Trials. 2021;22(1):661.

3. Watson-Jones D, Changalucha J, Whitworth H, Pinto L, Mutani P, Indangasi J, et al. Immunogenicity and safety of one-dose human papillomavirus vaccine compared with two or three doses in Tanzanian girls (DoRIS): an open-label, randomised, non-inferiority trial. Lancet Glob Health. 2022;10(10):e1473-e84.

4. Baisley KJ, Whitworth HS, Changalucha J, Pinto L, Dillner J, Kapiga S, et al. A dose-reduction HPV vaccine immunobridging trial of two HPV vaccines among adolescent girls in Tanzania (the DoRIS trial) - Study protocol for a randomised controlled trial. Contemp Clin Trials. 2021;101:106266.

5. Scherer EM, Smith RA, Gallego DF, Carter JJ, Wipf GC, Hoyos M, et al. A Single Human Papillomavirus Vaccine Dose Improves B Cell Memory in Previously Infected Subjects. EBioMedicine. 2016;10:55-64.

6. Kreimer AR, Rodriguez AC, Hildesheim A, Herrero R, Porras C, Schiffman M, et al. Proof-of-principle evaluation of the efficacy of fewer than three doses of a bivalent HPV16/18 vaccine. Journal of the National Cancer Institute. 2011;103(19):1444-51.

7. Safaeian M, Porras C, Pan Y, Kreimer A, Schiller JT, Gonzalez P, et al. Durable antibody responses following one dose of the bivalent human papillomavirus L1 virus-like particle vaccine in the Costa Rica Vaccine Trial. Cancer prevention research (Philadelphia, Pa). 2013;6(11):1242-50.

8. Safaeian M, Sampson JN, Pan Y, Porras C, Kemp TJ, Herrero R, et al. Durability of Protection Afforded by Fewer Doses of the HPV16/18 Vaccine: The CVT Trial. Journal of the National Cancer Institute. 2018;110(2).

9. Kreimer AR, Sampson JN, Porras C, Schiller JT, Kemp T, Herrero R, et al. Evaluation of durability of a single-dose of the bivalent HPV vaccine: the CVT Trial. Journal of the National Cancer Institute. 2020.

10. Tsang SH, Sampson JN, Schussler J, Porras C, Wagner S, Boland J, et al. Durability of Cross-Protection by Different Schedules of the Bivalent HPV Vaccine: the CVT Trial. Journal of the National Cancer Institute. 2020.

11. Tsang SH, Schiller JT, Porras C, Kemp TJ, Herrero R, Schussler J, et al. HPV16 infection decreases vaccine-induced HPV16 antibody avidity: the CVT trial. NPJ Vaccines. 2022;7(1):40.

12. Kreimer AR, Struyf F, Del Rosario-Raymundo MR, Hildesheim A, Skinner SR, Wacholder S, et al. Efficacy of fewer than three doses of an HPV-16/18 AS04-adjuvanted vaccine: combined analysis of data from the Costa Rica Vaccine and PATRICIA Trials. The Lancet Oncology. 2015;16(7):775-86.

13. Herrero R, Hildesheim A, Rodriguez AC, Wacholder S, Bratti C, Solomon D, et al. Rationale and design of a community-based double-blind randomized clinical trial of an HPV 16 and 18 vaccine in Guanacaste, Costa Rica. Vaccine. 2008;26(37):4795-808.

14. Paavonen J, Naud P, Salmeron J, Wheeler CM, Chow SN, Apter D, et al. Efficacy of human papillomavirus (HPV)-16/18 AS04-adjuvanted vaccine against cervical infection and precancer caused by oncogenic HPV types (PATRICIA): final analysis of a double-blind, randomised study in young women. Lancet (London, England). 2009;374(9686):301-14.

15. Sankaranarayanan R, Prabhu PR, Pawlita M, Gheit T, Bhatla N, Muwonge R, et al. Immunogenicity and HPV infection after one, two, and three doses of quadrivalent HPV vaccine in girls in India: a multicentre prospective cohort study. The Lancet Oncology. 2016;17(1):67-77.

16. Sankaranarayanan R, Joshi S, Muwonge R, Esmy PO, Basu P, Prabhu P, et al. Can a single dose of human papillomavirus (HPV) vaccine prevent cervical cancer? Early findings from an Indian study. Vaccine. 2018;36(32 Pt A):4783-91.

17. Basu P, Malvi SG, Joshi S, Bhatla N, Muwonge R, Lucas E, et al. Vaccine efficacy against persistent human papillomavirus (HPV) 16/18 infection at 10 years after one, two, and three doses of quadrivalent HPV vaccine in girls in India: a multicentre, prospective, cohort study. The Lancet Oncology. 2021;22(11):1518-29.

18. Joshi S, Anantharaman D, Muwonge R, Bhatla N, Panicker G, Butt J, et al. Evaluation of immune response to single dose of quadrivalent HPV vaccine at 10-year post-vaccination. Vaccine. 2023;41(1):236-45.

19. Gheit T, Muwonge R, Lucas E, Galati L, Anantharaman D, McKay-Chopin S, et al. Impact of HPV vaccination on HPV-related oral infections. Oral Oncol. 2023;136:106244.
